# In situ monitoring of *Lentilactobacillus parabuchneri* biofilm formation via real-time infrared spectroscopy

**DOI:** 10.1038/s41522-022-00353-5

**Published:** 2022-11-19

**Authors:** Diellza Bajrami, Stephan Fischer, Holger Barth, María A. Sarquis, Victor M. Ladero, María Fernández, Maria. C. Sportelli, Nicola Cioffi, Christine Kranz, Boris Mizaikoff

**Affiliations:** 1 Institute of Analytical and Bioanalytical Chemistry, Ulm University, Albert Einstein-Allee 11, 89081 Ulm, Germany; 2 Institute of Pharmacology and Toxicology, Ulm University Medical Center, Albert Einstein-Allee 11, 89081 Ulm, Germany; 3 Dairy Research Institute (IPLA-CSIC), Paseo Rio Linares s/n, 33300 Villaviciosa, Spain; 4 Chemistry Department, University of Bari ‘’Aldo Moro”, V. Orabona, 4, 70126 Bari, Italy

**Keywords:** Biofilms, Antimicrobials

## Abstract

Foodborne pathogenic microorganisms form biofilms at abiotic surfaces, which is a particular challenge in food processing industries. The complexity of biofilm formation requires a fundamental understanding on the involved molecular mechanisms, which may then lead to efficient prevention strategies. In the present study, biogenic amine producing bacteria, i.e., *Lentilactobacillus parabuchneri* DSM 5987 strain isolated from cheese were studied in respect with biofilm formation, which is of substantial relevance given their contribution to the presence of histamine in dairy products. While scanning electron microscopy was used to investigate biofilm adhesion at stainless steel surfaces, in situ infrared attenuated total reflection spectroscopy (IR-ATR) using a custom flow-through assembly was used for real-time and non-destructive observations of biofilm formation during a period of several days. The spectral window of 1700–600 cm^−1^ provides access to vibrational signatures characteristic for identifying and tracking *L. parabuchneri* biofilm formation and maturation. Especially, the amide I and II bands, lactic acid produced as the biofilm matures, and a pronounced increase of bands characteristic for extracellular polymeric substances (EPS) provide molecular insight into biofilm formation, maturation, and changes in biofilm architecture. Finally, multivariate data evaluation strategies were applied facilitating the unambiguous classification of the observed biofilm changes via IR spectroscopic data.

## Introduction

Biogenic amines are nitrogen organic compounds with low molecular weight that are of concern in food industries^1^. The consumption of foods with elevated content of biogenic amines could lead to a variety of intoxication symptoms posing a threat to consumer health^2^. The presence of biogenic amines also serves as a useful indicator of food spoilage e.g., due to the growth of decarboxylase-positive microorganisms^3^. Bacterial biofilms at industrially relevant surfaces act as reservoirs of contaminating microorganisms, which may lead to health concerns and issues with food safety. The intoxication with foodborne biogenic amines—and especially histamine—may lead to e.g., allergic reactions^4,5^. Several bacterial cultures and yeast present in food stuff are capable of decarboxylating amino acids, histidine, resulting in the formation of histamine^6,7^. Histamine is a heterocyclic amine with multiple toxicological effects^4^, and is rated since 2011 as a qualitative risk by the European Food Safety Authority with toxic effects in healthy individuals at concentrations >50 mg per person per meal^8^. The most notorious dairy food poisoning caused specifically by histamine requires strategies that prevent its synthesis, which in turn prerequisites knowledge on the mechanisms that regulate histamine production and metabolic pathways involved during its accumulation^2^. Gram-positive bacteria are the main producers of biogenic amines in dairy product industries with lactic acid bacteria (LAB) being the main histamine producers^2^. Different strains belonging to diverse species from the genus *Lactobacillus* have been reported as potential histamine producers in cheese during contact of food with equipment surfaces^5^. The dairy histamine-contaminating species *Lentilactobacillus parabuchneri* has been shown to form robust biofilms along with exceptionally high histamine production^9,10^. Production of histamine in *Lactobacillus* has been shown to occur at early growth stages^11^, and constitutes a system surviving in high acidic environments^10,12^. *L. parabuchneri* is a Gram-positive, facultatively anaerobic bacterium^9^ known as obligately hetero-lactic bacteria^13,14^. They are present in a wide variety of food products as biogenic amine-producing bacteria, which cause food poisoning resulting from biofilms accumulating histamine in cheese and other dairy products^15^. *L. parabuchneri* is an important part of the non-starter lactic acid bacteria community^16^. The presence of histamine-producing bacteria in foods is initially caused by food contamination from equipment surfaces during dairy production and post-ripening processing including but not limited to grating, slicing, and cutting^10,17^. Biofilms produced at food processing equipment surfaces are therefore considered the main ultimate source of histamine contamination with high developmental capability^9^. The dynamic process of biofilm formation by foodborne pathogens may subsequently lead to severe food spoilage resulting in foodborne illness^18^*. Lactobacilli* biofilms have been studied predominantly in the context of positive interactions serving as probiotic bacteria, whereas studies focused on food spoilage and contamination are scarce. The complexity of *Lactobacillus* biofilm formation—and the potential prevention—requires fundamental understanding on the associated molecular mechanisms. For developing efficient prevention strategies, it is essential identifying relevant molecular components and processes during early biofilm formation and their histamine-producing ability. Consequently, innovative analytical approaches facilitating biofilm activity studies are required. Ideally, the molecular properties of biofilms should be investigated during extended periods of time, yet, without physical disturbance. This requires in situ methods facilitating biofilm analysis in molecular detail at hydrated conditions.

IR spectroscopy has been proven as a useful analytical tool for gaining insight into processes and molecules related to biofilm formation close to real time via in situ analysis of hydrated biofilms^19,20^.

Attenuated total reflectance infrared spectroscopy (IR-ATR) is a highly suitable analytical method to obtain information on biofilm formation mechanisms, chemical properties, and changes in metabolic activity of surface-associated bacterial growth, if combined with appropriate microfluidics^21,22^. As microbial biofilms are communities of cells enclosed in self-produced extracellular polymeric (EPS) matrices adherent at surfaces, bacterial aggregates, and associated EPS are ideally studied via surface-sensitive—and surface-exclusive—analytical techniques such as IR-ATR evanescent field spectroscopy with the analytical signal probing few micrometers into the biofilm matrix established at a waveguide surface^23^. In situ IR-ATR analysis of microbial adhesion at biotic and abiotic surfaces enables monitoring molecular processes from early to late stages of biofilm formation^24^. For example, the contribution of EPS to the adhesion process during the early steps of cell adhesion was studied with bacteria deposited at the ATR waveguide surface^25^. At the solid-water interface, IR spectroscopy has facilitated the characterization of biofilms at a variety of substrates including natural solid surfaces at aquatic conditions and at bio surfaces such as lung epithelial cells and others^26^.

IR-ATR technologies are based on internal reflectance elements/waveguides (IREs) providing non-destructive probing of the chemical sample composition with high reproducibility^27^. The architecture and chemical properties of biofilms colonizing surfaces at the aqueous-phase–substrate interface during biofilm development was studied via IR-ATR spectroscopy in combination with optical microscopy^28^.

Biofilms grown directly at the ATR waveguide surface after inoculation with bacteria in a continuous flow system facilitates studying the evolution of biofilms over extended periods (i.e., days) in a continuous mode^29,30^. Of particular interest is access to molecular details such as the occurrence of extracellular proteins indicating colonization patterns^31^. Nascent biofilms consisting of a monolayer of microbial cells have been monitored at continuous flow conditions detecting the resulting spectral changes in response to modulated environmental parameters^32^. Exemplarily, it has been shown that free EPS (i.e., not attached to the cell surface) induces cell aggregation due to proteins harvested from media determining different growth phases^33^. Free EPS refers to polysaccharides, proteins, nucleic acid, and other biopolymers located outside the cell/bacteria, yet not directly attached to the biological surface. In contrast, EPS linked via covalent or noncovalent interactions is known as capsular (or cell-bound) EPS^34^. IR-ATR spectroscopy has also been used to determine biofilm constitution and extracellular matrix substances within the penetration depth of the evanescent field, i.e., extending a few micrometers from the ATR waveguide surface into the biofilm at flow conditions providing close to real-time, in situ information on native hydrated surface-attached biofilms^30,35^. Last but not least, using flow-cell assemblies that emulate natural flow conditions is relevant for comparing the obtained results to real-world dynamic scenarios^36,37^.

In the present study, the industrially relevant target bacteria *L. parabuchneri* DSM 5987 strain isolated from cheese was used as a model. Our study was focused on investigating their ability to form biofilms in molecular detail with the aim to understand the accumulation of histamine in processed cheese. To date, only few studies on the molecular mechanisms and properties of *L. parabuchneri* biofilms owning to the complexity of the associated microbial resources have been done^9,16,38,39^. Hence identifying relevant changes of molecular components during biofilm formation is key to developing efficient biofilm prevention strategies. Specifically, IR-ATR spectroscopy at flow conditions was applied for unraveling biofilm formation processes at yet unprecedented molecular detail during in situ monitoring of changes associated with molecular fingerprints of *L. parabuchneri* close to real time. It is anticipated that this fundamental concept will facilitate studies of more complex multi-phase/multi-species and disintegration processes via suitable antimicrobials.

## Results

### *Lentilactobacillus parabuchneri* in the planktonic state

**Fig. 1:**
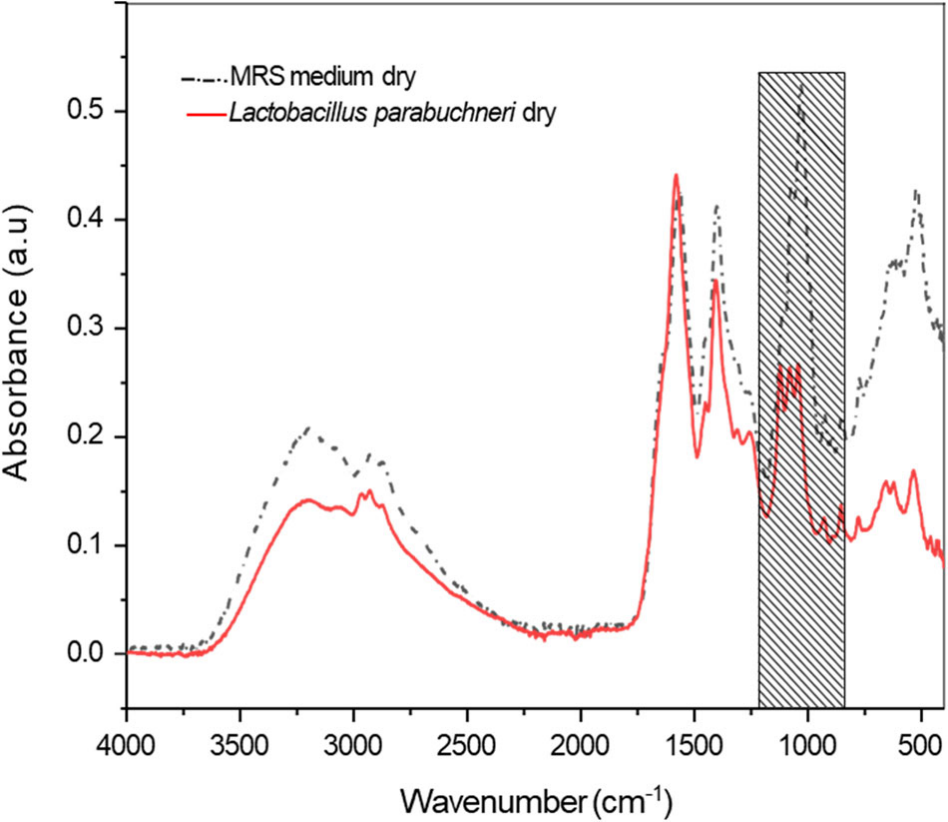
Infrared signatures of *L. parabuchneri* planktonic cells. Full-range IR-ATR spectra of dry planktonic *L. parabuchneri* (red spectrum) and dry MRS medium (dashed black spectrum).

**Fig. 2:**
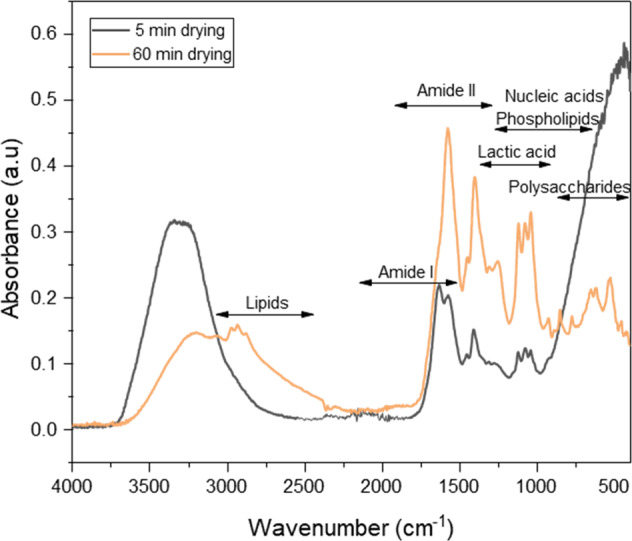
IR-ATR spectra of *L. parabuchneri* planktonic cells recorded during the drying process. IR-ATR spectra were recorded every 5 min until 60 min.

To confirm the findings on the behavior of *L. parabuchneri* biofilms and to access the development of bacterial communities, initially a spectrum of planktonic *L. parabuchneri* was recorded using a single-bounce diamond ATR assembly (Platinum ATR; Bruker Optik GmBH, Ettlingen, Germany) connected to an FT-IR spectrometer (Alpha I; Bruker Optik GmBH, Ettlingen, Germany). Evaporated MRS medium was recorded as a background spectrum. The IR spectrum of the planktonic form of *L. parabuchneri* was collected during 60 min of the drying process at environmental conditions. The obtained IR-spectra of *L. parabuchneri* planktonic cells were analyzed and revealed signatures characteristic for relevant molecular constituents including proteins, amides, lactic acid, nucleic acids, phospholipids, and polysaccharides^36,40^. Typical IR-spectra of planktonic *L. parabuchneri* are shown in Figs. 1 and 2. It is evident that the spectral regions of interest for the identification and discrimination of *L. parabuchneri* are 3000–2800 cm^‒1^,1800–1200 cm^‒1^, 1200–900 cm^‒1^, and 900–700 cm^‒1 ^^41^, respectively. The spectral window 3800–3000 cm^‒1^ represents the symmetric and asymmetric stretching modes of the OH bonds. The broad positive peak at 3400 cm^−1^ indicates water alongside *L. parabuchneri* planktonic cells, as shown in Figs. 1 and 2. Water at the waveguide surface is gradually replaced by planktonic bacterial cells becoming adherent, which is evident by a slight decrease in intensity of the hydroxylic group signatures^42^. In detail, the range 3000–2800 cm^‒1^ is characteristic for lipids or fatty acids of the bacterial cell membrane, 1800–1500 cm^‒1^ corresponds to the amide I and amide II bands resulting from proteins and peptides, 1500–1200 cm^‒1^ relates to spectral contributions by lactic acid, proteins and phospholipids, and 1200–900 cm^‒1^ results from EPS (shaded spectral region in Fig. 1) including predominantly polysaccharides convoluted with the fingerprint region of other molecular components, and is of particular interest indicating surface adhesion processes^43^. OH functional groups are present in the extracellular polymeric matrix due to the presence of carboxylic acids, alcohols, phenols, carboxylates and polysaccharides. In the region 1040–1080 cm^−1^, the stretching vibrations of OH-groups contribute to the ‘fingerprint’ of EPS associated with the bacterial cells^44^. Recording IR-ATR during the drying period reveals that the molecular features may be enhanced, if more material is accumulated close to the ATR waveguide surface^45^. It is evident that the spectral signatures of relevant molecular components including proteins, amides, lactic acid, nucleic acids, phospholipids, and polysaccharides appear more pronounced during that process.

### Early stages of biofilm formation: initial adhesion

The accumulation and ability of producing histamine from *L. parabuchneri* DSM 5987 strain results from the capability of decarboxylating the amino acid histidine. To investigate the effects of biofilm formation with respect to the levels of histamine, the following parameters were studied in detail. Firstly, the accumulation of microbial biomass at the IRE surface was immediately evident by the associated increase of related IR signatures. Secondly, during biofilm formation at polystyrene, stainless steel, metallic, and biotic/abiotic surfaces elevated CO^2^ levels and an increase in pH result from the decarboxylation of free amino acids resulting from histidine addition, which are main indicators of metabolic activity^13^. In addition, in contaminated cheese the increased amount of histamine results from the ability of the strain to decarboxylate histidine. At anoxic conditions, *L. parabuchneri* also shows the anaerobic conversion of lactate/lactic acid and the degradation of lactic acid in the presence of alternative electron acceptors such as glycerol, while producing acetate, 1–3 propanediol, CO_2_, and free short-chain fatty acids. The metabolic pathway reaction of the anaerobic conversion is shown as follows:

$$\begin{array}{*{20}{c}} {2\,{{{\mathrm{lactate}}}} \to 1,2 - {{{\mathrm{propanediol}}}} + \left| {{{{\mathrm{acetate}}}} + } \right|{{{\mathrm{CO}}}}_2} \\ {{{{\mathrm{lactate}}}} \to {{{\mathrm{lactic}}}}\,{{{\mathrm{acid}}}} \to {{{\mathrm{lactaldehyde}}}} \to 1,2{{{\mathrm{propanediol}}}}} \\ \downarrow \\ {{{{\mathrm{acetic}}}}\,{{{\mathrm{acid}}}}} \end{array}$$

This conversion is influenced by pH and temperature (e.g., during sterilization in an autoclave with acidic conditions required to induce lactate conversion)^46^. Anaerobic degradation of lactic acid by *L. parabuchneri* does not support cell growth. The metabolic pathway of *L. parabuchneri* is based on lactate production after primary fermentation of water-soluble carbohydrates, which leads to the acidity of the medium. The formation of fermented products and the increase of optical density because of bacterial growth is mainly resulting from the glucose as carbon-containing source within the MRS medium. The degradation of lactate occurs after this stage once most sugar is consumed, and the pH has decreased.

Changes in the IR-spectra reveal distinct differences in the microbial fingerprint as an indicator of attachment and colonization at the crystal surface. The process of *L. parabuchneri* biofilm formation at the ATR waveguide surface with the five distinct stages of biofilm development (Supplementary Fig. 2) supports the differences in adhesion properties. Consequently, an increase in nucleic acid and polysaccharide bands during the first hours of *L. parabuchneri* biofilm formation vs. the amide II band around 1577 cm^‒1^ associated with proteins is evident^47^.

**Fig. 3:**
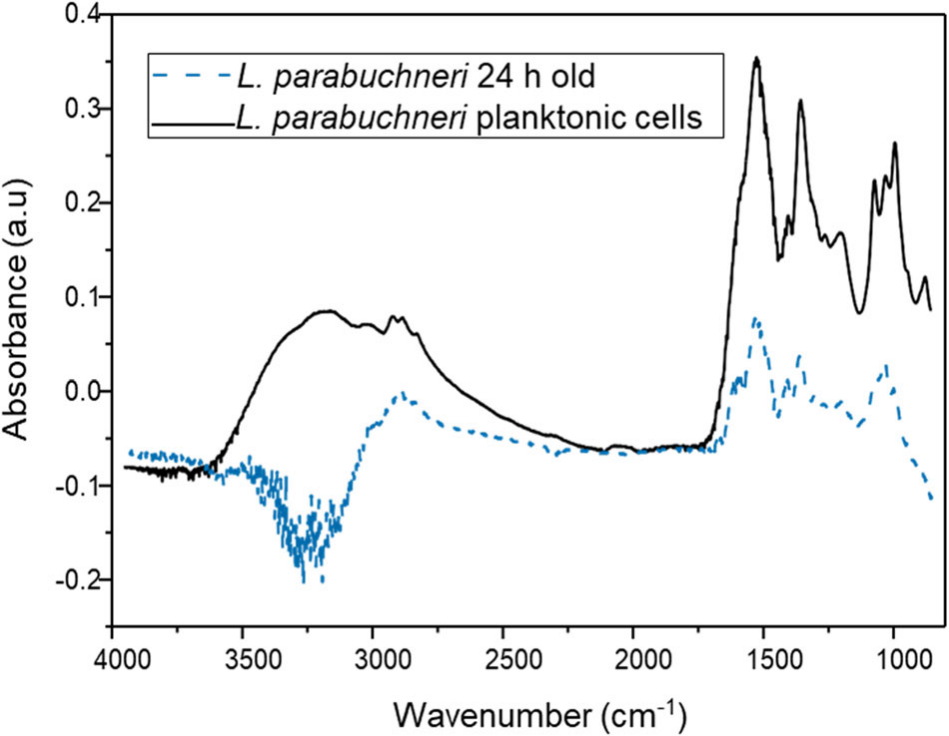
Molecular changes in the planktonic form of *L. parabuchneri* vs 24 old biofilms. Comparison of IR spectra of 24 h old *L. parabuchneri* biofilm performed in flow-through mode (black spectrum) vs. planktonic cells at static conditions (dashed blue spectrum).

Figure 3 shows a comparison of the IR-spectra of planktonic form of *L. parabuchneri* vs. the spectrum of a 24 h old *L. parabuchneri* biofilm. Both spectra were normalized to the amide II band, which is less affected by the water background^36,37^. MRS medium was collected as the background spectrum; therefore, negative bands are observed once bacteria become adherent and displace MRS from the analytically probed volume adjacent to the waveguide surface. The observed somewhat less resolved IR signatures relate to the flow-through conditions vs. static analysis of planktonic cells.

Evidently, planktonic cells produced more pronounced IR-signatures compared to bacterial biofilms, which is attributed to the IR measurements executed at static vs. flow-through conditions for biofilm studies. Still, all relevant bands (e.g., indicative of phospholipids and proteins related to bacterial membranes at 1449 cm^‒1^ and 1260 cm^‒1^, respectively) are distinctly evident^48^.

**Fig. 4:**
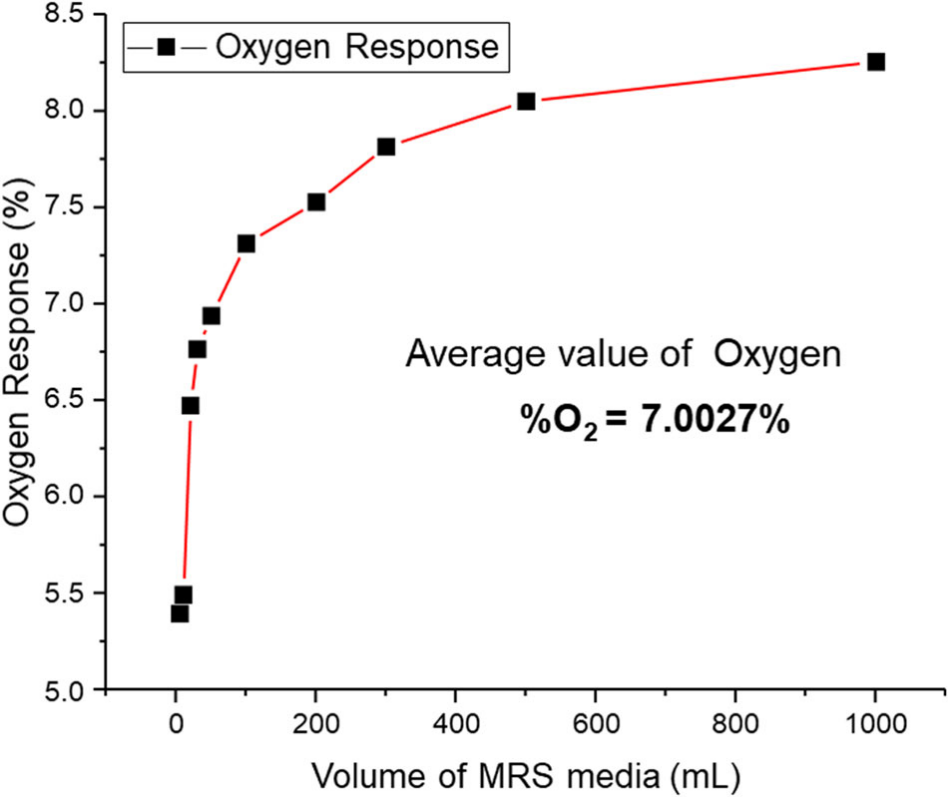
Oxygen monitoring in growth media. The obtained oxygen levels are shown as response of the fiberoptic oxygen probe (%) vs. mL of MRS medium.

Lactic acid bacteria adapt their respiration capacity upon oxygen variations (e.g., gradients in the microaerobic range)^49,50^. Since *L. parabuchne*ri are anaerobic aerotolerant bacteria, prior to the inoculation of the suspension, a microaerophilic environment was ensured by purging the growth medium with nitrogen facilitating continuous bacterial growth. Figure 4 and Table 1, summarize a typical oxygen trace monitored via a luminescence-quenching-based optical oxygen probe during the degassing procedure considering that the oxygen tolerance in anaerobic bacteria such as *L. parabuchneri* permits 5–10% such that microbial growth is not inhibited^1^.

**Table 1:** Oxygen values determined in degassed MRS media.

Time of degassing (min)	Volume of MRS media (mL)	Oxygen response (%)
4	5	5.39
7	10	5.49
10	20	6.47
12	30	6.76
14	50	6.94
15	100	7.31
17	200	7.53
20	300	7.81
25	500	8.05
30	1000	8.25

### Long-term monitoring of *L. parabuchneri* biofilm formation at flow conditions

**Fig. 5:**
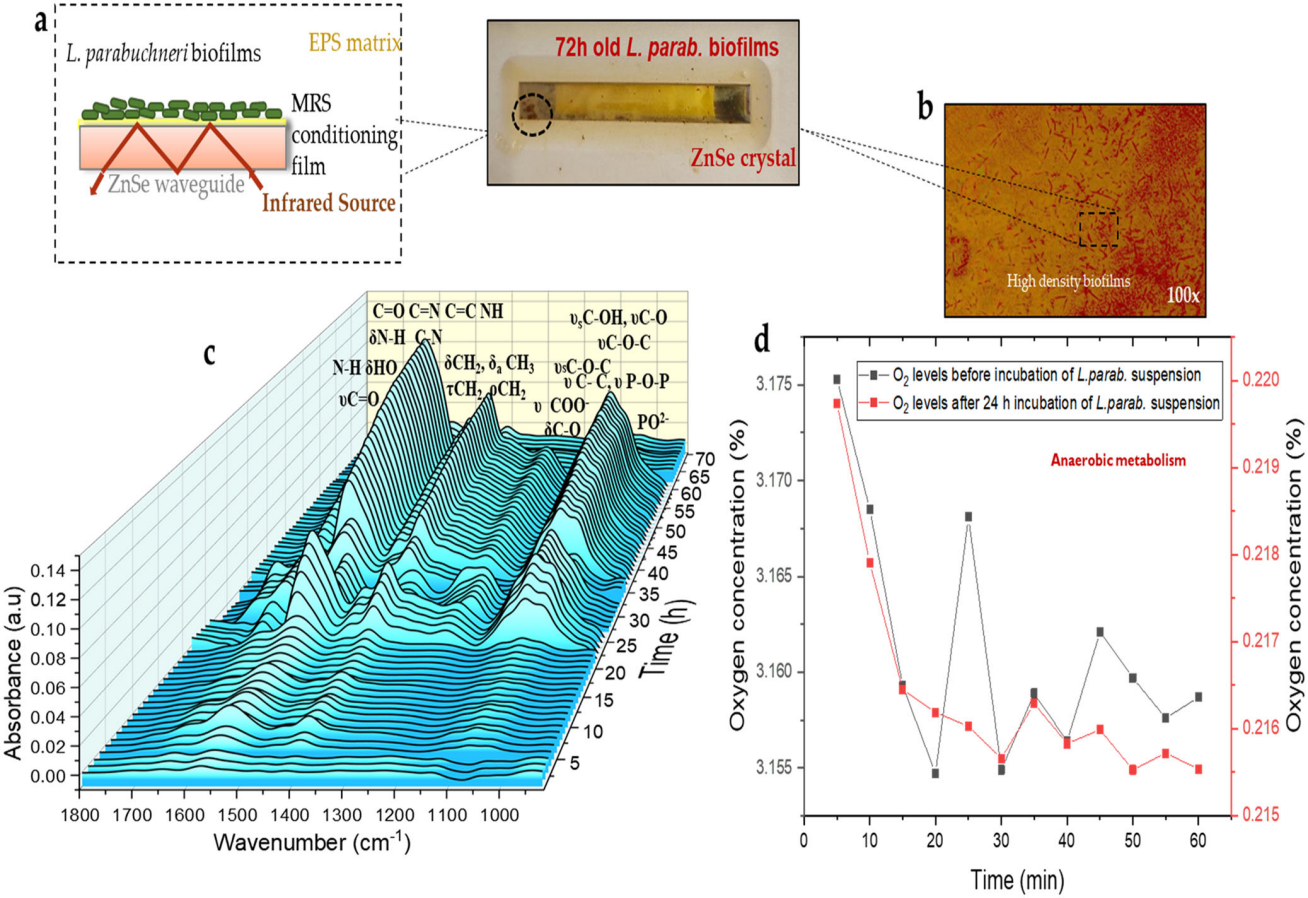
Biofilm evolution. **a** Scheme of the deposition of *L. parabuchneri* biofilms at the ATR waveguide surface after the conditioning film was formed. **b** Optical image of *L. parabuchneri* bacterial biofilm attached to the surface of the ATR waveguide during 7 days of IR measurements at flow-through conditions. **c** 3D plot of IR-ATR spectra vs. time revealing the molecular changes occurring during 72 h of biofilm development. **d** Concentration profiles of oxygen prior to and after inoculation of the *L. parabuchneri* suspension.

In the following, the formation of *L. parabuchneri* DSM 5987 strain biofilm was evaluated through the biomaterial that is deposited at the surface of the ATR crystal. IR-spectra were obtained right after inoculation using the IR-ATR flow cell assembly and recorded continuously during and after periods up to 72 h. Prior to the inoculation of *L. parabuchneri* suspension, a conditioning film was developed by flushing anaerobic sterile MRS medium for 120 min across the waveguide surface facilitating microbial adhesion. This MRS conditioning film prevents the attachment of *L. parabuchneri* directly at the ZnSe crystal surface, which may have potential detrimental effects on bacterial cells. The bacterial suspensions contained bacteria that have already reached the end of their exponential growth phase at 1.16 × 10^9^ cells/mL (OD_600_ = 1.45) and 1.38 × 10^9^ CFU/mL (OD_600_ = 1.73). The number of viable cells in the bacterial culture was determined previously using the serial dilutions method^51^ by picking a colony of *L. parabuchneri* bacteria. The corresponding changes of the IR bands related to biomass accumulation at the IRE surface were observed predominantly via the amide bands, which are an indicator of biogenic amine (BA) producing bacterial histamine release present in biofilms covering the ATR crystal surface^36^. The temporal evolution of the IR-spectra is shown in Fig. 5c. evidencing the increase in characteristic bacterial growth IR signatures.

The chemical behavior of living cells^52–54^ via in situ, real-time IR spectroscopy allows unraveling mechanisms involved in biofilm-surface interactions. The most relevant signatures are found in the spectral region from 1800 to 600 cm^‒1^. Specifically, the bands at 1577 cm^‒1^ and 1567 cm^‒1^ may be attributed to the N‒H bending, C–N stretching, and asymmetric stretching band for deprotonated COO^‒^, i.e., the strongest bands within the amide I and amide II regime arise from overlapping νC = O/δN−H, and δN−H/νC−N vibrations^37^. The peaks observed at 1353 cm^‒1^ and 1450 cm^‒1^ are assigned to the bending modes of -CH_3_ and -CH_2_ associated with proteins (δCH_2_, δCH_3_), while the peak at 1401 cm^‒1^ result from the symmetric C‒O stretching vibration of the carboxylate groups (υ_sym_COO^‒^). The stretching P=O vibration derives from the polyphosphate and phosphodiester products, and appears around 1081 cm^‒1^^48,55^. The symmetric stretching vibration of the phosphoryl groups provided information on the presence of extracellular polymeric substances (EPS); the broad and intense band at 1160–1003 cm^‒1^ includes a combination of symmetric stretching vibrations resulting from υC–O, υC–O–C, and υP–O–C groups^25^.

**Fig. 6:**
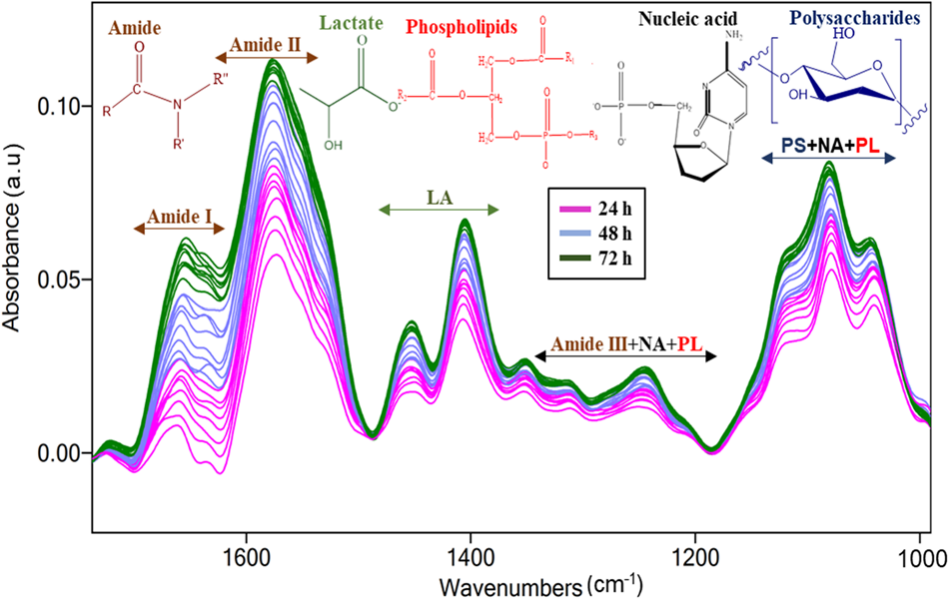
IR-ATR spectra of *L. parabuchneri* biofilms monitored for 24, 48, and 72 h during real-time incubation within the flow system. The observed spectral changes correspond to proteins (amide I: 1700–1618 cm^−1^, amide II: 1585–1486 cm^−1^), LA: 1465–1293 cm^−1^, amide III + NA + PL: 1350–1180 cm^−1^ and PS + NA + PL: 1189–960 cm^−1^, respectively (LA = lactic acid or lactate, NA = nucleic acids, PL = phospholipids, PS = polysaccharides).

The microbial decarboxylation pathway of histidine in a medium of low pH^56,57^ results in histamine and is specifically induced via aggregations of *L. parabuchneri* embedded in EPS. The operation of *L. parabuchneri* biofilms rich with EPS matrix at the ATR waveguide surface after the conditioning film was formed is schematically illustrated in Fig. 5a). The process of *L. parabuchneri* biofilm formation at the ATR waveguide surface reveals five well-defined stages of biofilm evolution^58,59^ (see suppl/ material). Adhesion is initiated via reversible and non-reversible attachment, which is driven by van der Waals and electrostatic forces responsible for interaction of the surface with host cells^60^. Bacteria affect this process via the expression of molecules mitigating binding between the substrate and the EPS matrix^61^. The transport of planktonic *L. parabuchneri* bacteria through suspended species from the MRS medium and the initial layer of bacteria adherent at the waveguide surface is affected by the attachment of the species to the conditioning film (see Fig. 5a). Bacterial cells adherent to the conditioning film provides a layer rich in nutrients. Depending on the surrounding medium, the conditioning layer of media consists of relevant trace elements for cell attachment such as glycoproteins, complex polysaccharides, and humic compounds. Herein, the chemistry and the architecture of the biofilms were evaluated by a combination of IR-ATR spectroscopy and light microscopy. After 7 days of inoculation with the ATR flow assembly, the ZnSe surface was distinctly covered with colonizing *L. parabuchneri* bacteria responsible for a high-density biofilm yet heterogeneously distributed bacterial microcolonies (see Fig. 6a, b). The assignment of the spectral contributions tracks the biomolecules related to proteins (i.e., nucleic acid and phospholipids). Spectral features of polysaccharides were more pronounced for older biofilms (Fig. 5c). Lactic acid production varied with time while the first layers of biofilm were deposited at the ZnSe waveguide surface within the penetration depth of the IR radiation^36^.

The quantitative determination of oxygen is an important parameter in food packaging, bioprocess control, and industrial production monitoring. The integration of optical oxygen sensors providing readings in the liquid and the gas phase facilitated measurements that are not influenced by the flow rate of the sample with excellent long-term stability. The oxygen trace was measured inside the flow cell next to the ATR waveguide surface. As the biofilm thickness is increased due to the proliferation of the microorganisms, the reduction in oxygen level at the base of the *L. parabuchneri* biofilm (i.e., close to the ATR waveguide surface) may be attributed to associated oxygen diffusion limitations with increasing biofilm thickness (see Fig. 6d). The decrease of the oxygen concentration was noticed prior to the inoculation of *L. parabuchneri* bacterial suspension into the flow system, and after inoculation for controlling the biofilm growth. The latter was determined via a luminescence sensor positioned within the *L. parabuchneri* biofilm.

Due to the production of lactic acid, a decrease in pH to a value of ~4.5 occurs, and this acidic environment leads to physico-chemical changes in the external layer of peptidoglycans at the bacterial cell wall. This in turn leads to more IR-spectral fingerprints of polysaccharides within increasingly matured biofilms^62^. With increasing thickness of biofilm, the layer adjacent to the ATR waveguide surface will suffer from a diffusion-limited deficiency in nutrients evidenced by a slight decrease in IR band intensities for the amide I and II features, while the other IR bands remain constant. Sterile medium continues to deliver nutrients to the biofilm-entrapped community, thus stimulating further biofilm growth, as evident by the increased intensity of IR bands in the period after supply of fresh MRS medium (see Figs. 6 and 9). Dispersal and detachment of mature biofilm clusters^58,63,64^ results in *L. parabuchneri* cell transfer from the biofilm matrix to the bulk liquid serving as a survival mechanism promoting transfer into new habitats. The dispersed cells enter into a new cycle of biofilm formation and facilitate once more reproduction^65^. The evaluation of *L. parabuchneri* biofilms by assignment of characteristic IR bands during several days of real-time monitoring is based on comparing changes in relative intensities of representative IR signatures (Fig. 6). The increase in acidity of the media is evidenced by lactic acid production associated with the features at 1405, 1313, and 1250 cm^−1^, respectively as a byproduct of anaerobic respiration^36^. During long-term measurements, increasing IR signatures of polysaccharides, nucleic acids, and phospholipids bands reveal more biomass via peptides and protein chains associated with the bacterial cell membrane.

IR-ATR spectra of *L. parabuchneri* biofilms monitored for three consecutive days reveal chances corresponding to proteins (amide I: 1700–1617 cm^‒1^, amide II: 1577–1464 cm^‒1^), lactic acid/lactate: 1465–1293 cm^‒1^, nucleic acids + phospholipids: 1280–1180 cm^‒1^ and polysaccharides + nucleic acids + phospholipids: 1121–997 cm^‒1^. This demonstrates that the spectral range 1700–600 cm^‒1^ is uniquely suitable for the identification of *L. parabuchneri* biofilms providing access to adhesion mechanisms of cells attached to the substrate and physical-chemical changes during extended biofilm growth periods.

In IR-ATR spectroscopy, IR radiation is coupled into a high-refractive-index waveguide (a.k.a., internal reflection element; IRE) in contact with a lower-refractive-index sample, such as the bacterial solution or the biofilm and propagates below the so-called critical angle via total internal reflection along the waveguide^66^. At each reflection at the IRE/sample interface, an evanescent field is generated leaking into the sample matrix facilitating evanescent field absorption spectroscopy recording the infrared fingerprint of sample similar to a conventional transmission-absorption experiment^42^. However, in the latter case strongly absorbing media such as water limit to few tens of micrometers of transmission pathway and does not allow for studying surface processes such as bacterial adhesion, which remains exclusive to IR-ATR spectroscopy. The evanescent field penetrates with exponentially decaying intensity a few micrometers (i.e., at the penetration depth; *d*_p_) into the adjacent sample medium^67,68^, i.e., here the biofilm. With knowledge on the refractive index of the crystal *n*_c_ and of the sample *n*_s_ (with *n*_c_ > *n*_s_), the penetration depth of the evanescent field can be approximated by^27,69^:

1 $$d_{\rm{p}} = \frac{\lambda }{{2\pi \left( {n_{\rm{c}}^2\mathop {{\sin }}\nolimits_\theta ^2 - n_{\rm{s}}^2} \right)^{1/2}}}$$

with *λ* as the wavelength and θ the incidence angle at the IRE/sample interface.

The MIR spectral regime is characterized by a relatively high penetration depth *d*_p_, as it benefits from the long wavelength largely dominating *d*_p_, which defines the probed analytical volume. ZnSe is a commonly used MIR-transparent IRE material with a refractive index of 2.4^70^. Hence, the penetration depth *d*_p_ exemplarily calculated at *λ* = 10 μm (1000 cm^−1^) for the ZnSe IRE (*n*_c_ = 2.4) and the refractive index of the biofilm (~1.4) at an angle of incidence *α* = 45° amounts to ~1.6 μm^31,71^. Therefore, only bacteria and processes in close vicinity or at the ATR waveguide surface are probed via evanescent field absorption spectroscopy considering that the average penetration depth in the range of few micrometers^72^. The dynamic examination of base layer films provides information on the contact area between ATR waveguide and the biofilm. Hence, the results reported herein pertain to few—albeit probably the most important—microbial layers at the surface averaging information in the x,y-plane, while omitting bulk information in z-direction.

### Unraveling molecular details via deconvolution of IR-ATR spectra

IR spectra representing the complex structures of microbial biofilms require advanced data processing strategies during peak deconvolution given broad and overlapping combination and overtone absorption peaks. This prerequisite a detailed analysis and comprehensive characterization of the biochemical features with respect to overlapping IR features owing to the complexity of the bacterial matrix. Cumulative fit functions are among the most commonly used methods for processing complex IR spectra enabling a spectral decomposition via appropriate curve-fitting algorithms^73,74^. Yet, it should be noted that this strategy requires a detailed IR-spectroscopic characterization of the individual molecular components for accurately identifying the characteristic peak positions prior to deconvolving mixture spectra. to specific individual peaks, simplifying the influence of nutrient components for biofilm investigation. While there are several approaches for spectral deconvolution considering the shape of the peaks^75^, in case of IR-ATR spectra, overlapping or broadened signals from different simultaneously absorbing cellular components^76^ were most accurately resolved via a Gaussian curve fit strategy.

For a more detailed analysis of the rather complex IR spectra, first the spectrum of MRS medium has been studied in detail via a so-called cumulative impulse fit for understanding the contribution of each individual molecular component to the observed spectral sum signature. Thereby, each component is individually spectroscopically characterized (Supplementary Fig. 3), and then theoretically fitted with—here Gaussian—peaks considering spectral position and band shape. Thereafter, one may add up the contributions and use a cumulative impulse fit to emulate the sum spectrum of all components vs. the experimentally obtained spectrum of MRS. This procedure enables precisely recognizing which of the molecular components within MRS are consumed or changed during the metabolic activities of bacterial cells at any given stage during biofilm maturation.

**Fig. 7:**
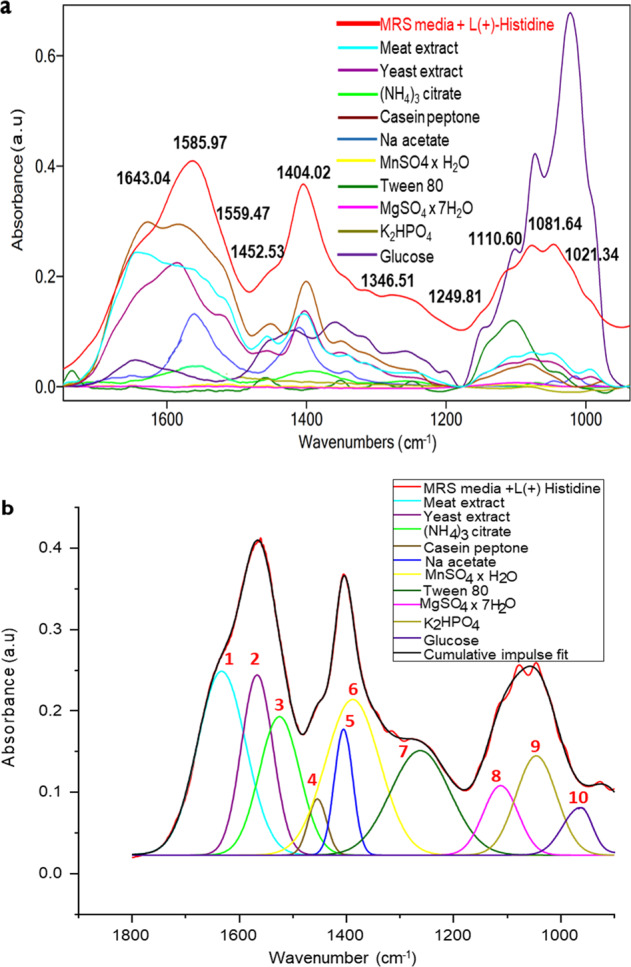
Deconvolution of IR spectra for unraveling molecular details during biofilm formation. **a** IR-ATR spectrum of MRS medium supplemented with 5 mM L ( + ) L-histidine (red) and experimentally obtained spectra of the individual constituents in the spectral region from 1800 to 900 cm^−1^ (see color code, top, middle). **b** Resulting cumulative impulse fit (black) and fitted IR signatures of the individual constituents (see color code, top, right). The integration values (peak center and width) of the individual components (see Table 2) served as restrictions for the cumulative impulse fit (*n* ≥ 10; Gaussian fit). The experimentally obtained sum spectrum is given in red in (**a**) and (**b**).

The initial deconvolution of MRS medium IR spectra via a so-called cumulative impulse fit is a fundamental prerequisite for the later analysis of *L. parabuchneri* biofilms within that culture medium. Figure 7a illustrates the spectrum of MRS in the spectral region of 1800–900 cm^−1^. Prior to this, the individual constituents of MRS media were IR-spectroscopically characterized for identifying their characteristic spectral individual spectral features and peak intensities. The characteristic peaks of the individual constituents were integrated and averaged, and the calculated peak height (H, a.u.), peak width at half height (w, cm^−1^) and peak center (x_0_, cm^−1^) was used as the basis to calculate the mean of at least 10 experimental datasets for the cumulative peak fit via Gaussian curves (*n* = 10). The parameters w and x_0_ were fixed at the calculated values, and the peak height was restricted to defined maximum values for avoiding over-parameterization and negative peaks, and to match the experimentally obtained peak heights as observed during recording individual MRS constituent spectra. The cumulative impulse fit performed on this experimentally derived basis then converged to an excellent chi-squared value of 8.75872 × 10^−6^.

The integration results are provided in Table 2 and served as the basis for the cumulative impulse fit shown in Fig. 7b. Considering the spectral resolution and the band shape, the MRS media with early-stage *L. parabuchneri* biofilms are characterized by a broad band indicating convoluted peaks from the media components. Yet, detailed analysis of the spectral features of MRS media (see Fig. 7a, b) now provides access to the spectral response initiated by the *L. parabuchneri* biofilm formation. The comparison of the individual constituent spectra in Fig. 7a with the individual Gaussian curves in Fig. 7b indeed reveals an excellent match. The convergence of the fit is corroborated by the corresponding minimal residual intensities in the range of 0.0002–0.2981 a.u., and further substantiated via the calculated values summarized in Table 2. Figure 7a shows the experimentally obtained IR spectrum of MRS (red) augmented by the individual experimental constituent spectra facilitating allocating the deconvoluted individual contributions of the molecular components color-coded in Fig. 7b along with the correspondingly resulting fitted sum spectrum (black). It is clearly evident that this reconstructed spectrum (black) obtained via cumulative impulse fitting resembles the experimentally obtained MRS spectrum (red) remarkably well. As shown, the MRS medium was supplemented with 5 mM L (+) L-histidine and the spectrum was obtained prior *L. parabuchneri* biofilm formation.

**Table 2:** Peak attributions of the mean values of individual MRS constituents evaluated for the cumulative impulse fit obtained via flow-through IR-ATR studies along with the statistical result of the Gaussian cumulative impulse fit.

	MnSO_4_ xH_2_O^78^ (*n* = 10)	Casein peptone^77^ (*n* = 10)	Na acetate^81^ (*n* = 10)	MgSO_4_ x7H_2_O^78^ (*n* = 15)	Meat extract^80^ (*n* = 10)	(NH_4_)_3_ citrate^80^ (*n* = 10)	Tween 80^37,80^ (*n* = 10)	Glucose^76^ (*n* = 10)	K_2_HPO_4_^79^ (*n* = 13)	Yeast extract^36^ (*n* = 10)
	ν_as_ (SO_4_)^2−^	(_υ_C=O); N–H	ν_as_ COO^-^	ν_as_ (SO_4_)^2−^	ν_as_ (C=O)	ν_as_ (C=OO-)	_as_ (C=C)	ν (C–O)	ν_as_ (PO)_4_^3-^	ν_as_ (C=C)
		δH_2_O								
x_0_ [cm^−1^]	1098.0	1626.73	1560.49	1110.60	1643.04	1559.47.8.	1104.91	1021.34	1081.6	1585.97
w [cm^−1^]	63.1	75.52	20.9323	68.2	67.96	62	9.06	91.09	44.9	53.55
H [a.u.]	0.0065	0.2981	0.13254	0.0248	0.14275	0.03713	0.11941	0.678	0.06735	0.12335
	ν_as_ (SO_4_)^2−^	(CH_2_)	(CH)	ν_as_ (SO_4_)^2−^	ν_as_ (-C=OO-)	ν (-CO_2_),	ν (C=O)	(OH)	ν_as_ (PO)	ν_as_ (-C=OO-)
x_0_ [cm^−1^]	1102.3	1452.53	1401.7	1102.35	1567.6	1399.8	1458	1347.8	991.5	1567.6
w [cm^−1^]	63.1	3.9	24.8	63.1	55.6	9.26	4.1	0.96	24.8	55.6
H [a.u.]	0.0004	0.09865	0.06135	0.0003	0.07995	0.04275	0.0208	0.0834	0.0321	0.07625
	ν_as_ (S=O)	ν(COO-), (OH)	ν(C–O)		(CH_2_)	(C–O)	ν(C–O) strong	υC–C, υP–O–P		_as_ (OH)
x_0_ [cm^−1^]	1346.5	1399.52	1012		1452.5	1107.5	1249.81	990.06		1401.14
w [cm^−1^]	2.7	6.0	12.8		33.9	64.7	14.8	11.5		55.6
H [a.u.]	0.0002	0.15482	0.01318		0.01885	0.0082	0.01976	0.06175		0.08285
Red.Chi-Square	8.75852 × 10^−6^		Gaussian Model							
R^2^	0.99904		y = y0 + A/(w*sqrt (pi/(4*ln (2)))) * exp(−4*ln(2)*(x-xc)^2/w^2)							

The peaks of the individual constituents show the dominating contributions of single components with the specific vibrations related to the molecule vibrations as presented in Table 1, and especially the bending vibration of the NH_2_ side chain groups of the inoculated MRS medium (MRS + *L. parab.*) as histamine production kicks in (Fig. 7a, b).

The asymmetric and symmetric features of the side chain groups resulting from the L(+)-histidine supplement within the MRS medium show characteristics of the individual MRS constituents. The protein content derived from the vibrational signatures of nucleic acids and phospholipids associated with casein peptone, yeast and meat extract exhibits valuable information once deconvoluted. The C=O, N–H asymmetric stretching vibrations at 1626 cm^‒1^ and symmetric bending vibrations of CH_2_ groups at 1626 cm^‒1^ are characteristic of casein peptone^77^, while S=O asymmetric stretching vibrations at 1346 cm^‒1^ are derived from MnSO_4_ x H_2_O. The asymmetric stretching ν_as_ (SO_4_) at 1110 cm^‒1^ is associated with MgSO_4_ x 7H_2_O^78^ as an inorganic salt present at low concentrations within the MRS broth. The symmetric and asymmetric P=O stretching vibrations at 1081 cm^‒1^ and 991 cm^‒1^ relate to K_2_HPO_4_^79^, and the polysaccharide or carbohydrate signals are associated with glucose^76^ (i.e., the C‒O stretching vibration at 1021 cm^‒1^). They are characteristic for the carbon source as the relevant nutritive component for cell growth. Among others, the cumulative impulse fit also reveals the prevailing contributions of the asymmetric stretching of COO- at 1559 cm^−1^ and of the symmetric stretching of -CO_2_ at 1399 cm^−1^ associated with ammonium citrate^80^. The bending vibration of CH at 1401 cm^−1^ is characteristic of sodium acetate^81^, the strong CO stretching vibration of polysorbate (Tween 80), and the symmetric stretching vibration of the carbonyl CO band^80^ at 1643 cm^−1^ relates to meat extract, while the 1585 cm^−1^ of the C=C asymmetric stretching derives from yeast extract (Table 1). Changes of the peak areas of the amide 1 band (1700–1617 cm^‒1^), amide II band (1577–1464 cm^‒1^), amid III band (1350–1200 cm^‒1^), nucleic acids (1280–1180 cm^‒1^)^82^ and the extracellular polymeric substances (1121–997 cm^‒1^)^25^ are distinct indicators of bacterial attachment and associated biofilm maturation. Absorptions of nucleic acids are expected in the spectral window 1350–1180 cm^−1^ of the band assigned to the functional groups >C=O, >C=N, >C=C< as stretching features of the DNA or RNA heterocycle base structures^83^. Extracellular DNA is required for bacterial biofilm formation^84^. On the other hand, the amide III peaks result from the combination of in-phase N=H in-plane bending with C–N stretching and C–H/N–H deformation vibrations. The amide III band is generally present with weaker intensity^85^. When attempting to separate the contributions of nucleic acids and the amide III band, it should be noticed that IR signals from nucleic acids and phospholipids overlap in the IR spectrum therefore masking the lower-intensity amide III signal^31^. Detailed IR vibrational peak attributions of *L. parabuchneri* constituents are shown in Supplementary Table 1.

**Fig. 8:**
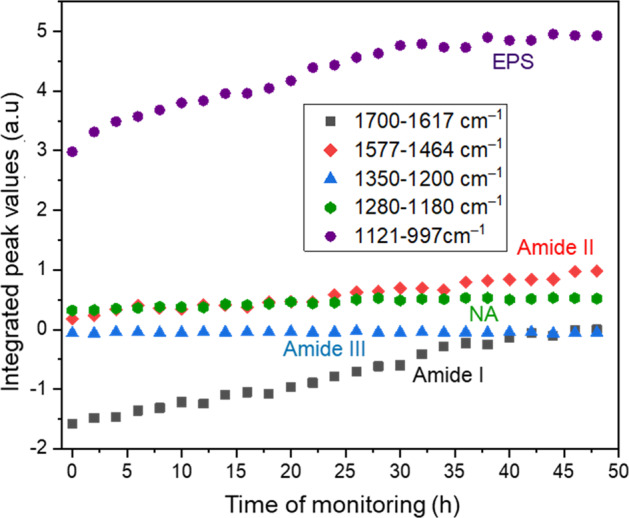
Temporal progression of characteristic integrated peak areas during 48 h of *L. parabuchneri* inoculation. (Reference spectrum: MRS medium (0.5 g L^−1^). Integrated peak areas for amide 1 band (1700–1617 cm^‒1^, black); amide II band (1577–1464 cm^‒1^, red); amid III band (1350–1200 cm^‒1^, blue); nucleic acids (1280–1180 cm^‒1^, green), and the extracellular polymeric substances (1121–997 cm^‒1^, purple) are shown.

Correspondingly, these bands are only observed during the period of *L. parabuchneri* cells inoculated into the ATR flow assembly, i.e., during 48 h of biofilm formation (Fig. 8). It is evident from the IPVs following the progression of the evaluated associated IR signatures that the levels of EPS significantly yet rather continuously increase more frequently than the amide I features, while the amide II band shows periodic fluctuations while increasing as a function of time corresponding to increasing protein levels. Amide III and nucleic acid features are not significantly increased during 48 h of real-time monitoring of *L. parabuchneri* biofilms but remain rather constant (Fig. 8).

The level of nucleic acids is indicated by the extracellular DNA and RNA band that is continuously increasing with time following the trend as the lipid and amide III profile comparable to polysaccharides during the initial bacterial attachment phase (see Fig. 8). As shown by Humbert et al. via IR studies, different adhesion kinetics were observed indicating potential changes in culture conditions, growth phase properties, and number of bacteria inoculated into the flow system^42^.

### Multivariate data evaluation

**Fig. 9:**
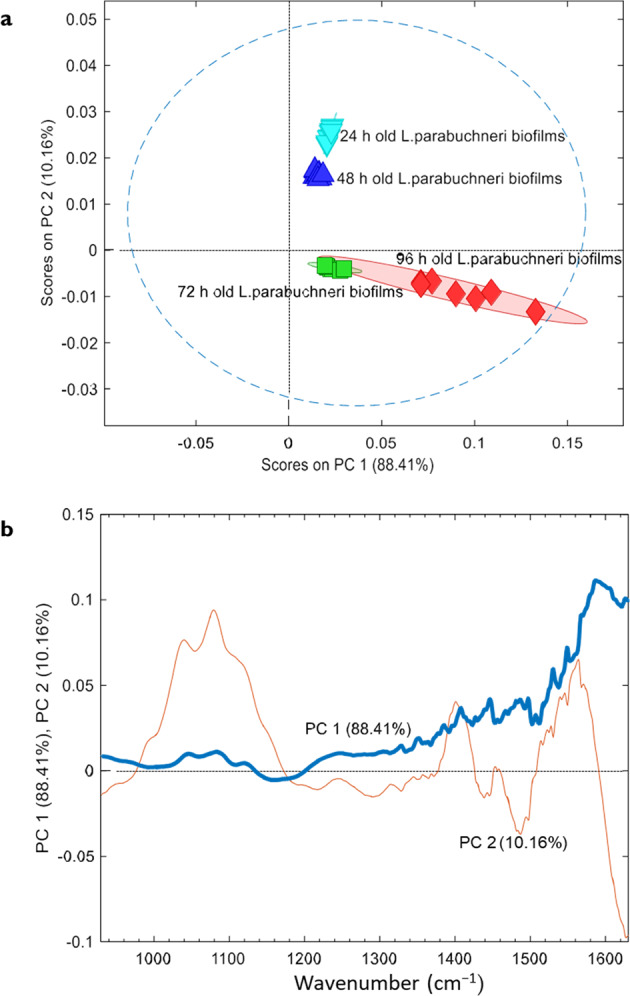
Multivariate classifications of IR spectral datasets of *L. parabuchneri* biofilms via principal component analysis. **a** Scores plot of the classified spectral data recorded after 24, 48, 72, and 96 h via PCA revealing that the obtained spectra clearly cluster along the time axis and that the recorded IR spectra unambiguously identify the maturation stage of the biofilm; **b** loadings of the two PCs explaining >98% of the total variance.

Multivariate data mining routines such as principal component analysis (PCA) have been used facilitating an alternative more comprehensive classification of the obtained IR data taking the entire spectra into account rather than deconvoluting into individual molecular components. PCA uses a transformation of a complex dataset (i.e., singular value decomposition) to convert a set of observations—here, IR spectra—into a coordinate system of orthogonal Eigenvectors in the variance data space^86,87^. This allows for rapidly classifying data according to the occurring variances, i.e., in the case of biofilm-associated IR spectra for which stage of biofilm formation the entirety of molecular changes is indicative of. Total variability of the independent variables is the criteria for calculation of principal component scores^88^. IR-data of *L. parabuchneri* biofilms collected during a period of 4 days were evaluated and classified via PCA into the stage of maturation (i.e., 24, 48, 72, and 96 h old). The scores plot (see Fig. 9a) clearly shows distinct data clusters enabling the unambiguous assignment of the age of the biofilm.

The discriminatory nature of the collected IR-spectra reflects in fact that only two latent variables (a.k.a., principal components) explain 98.57% of the total variance, and lead to a clear separation into clusters of spectra along the temporal evolution and maturation of *L. parabuchneri* biofilms. The PCA loading plot presented in Fig. 9b shows a notable peak in the polysaccharide region (1081 cm^−1^) which is basically referred to ν(C–O) coupled with δ(C–O) of C–OH groups of polysaccharides as one of the main components of biofilms. Prior to the data analysis, data pretreatment was executed calculating the first derivative of each spectrum using a Savitzky-Golay algorithm with nine smoothing points such that baseline shifts are minimalized. In future, such classification models will enable categorizing biofilms via IR-spectra according to their growth state, state of maturation, and—after appropriate studies—disintegration upon interaction, e.g., with antimicrobials.

### *L. parabuchneri* as moderate biofilm producer at stainless steel surfaces

**Fig. 10:**
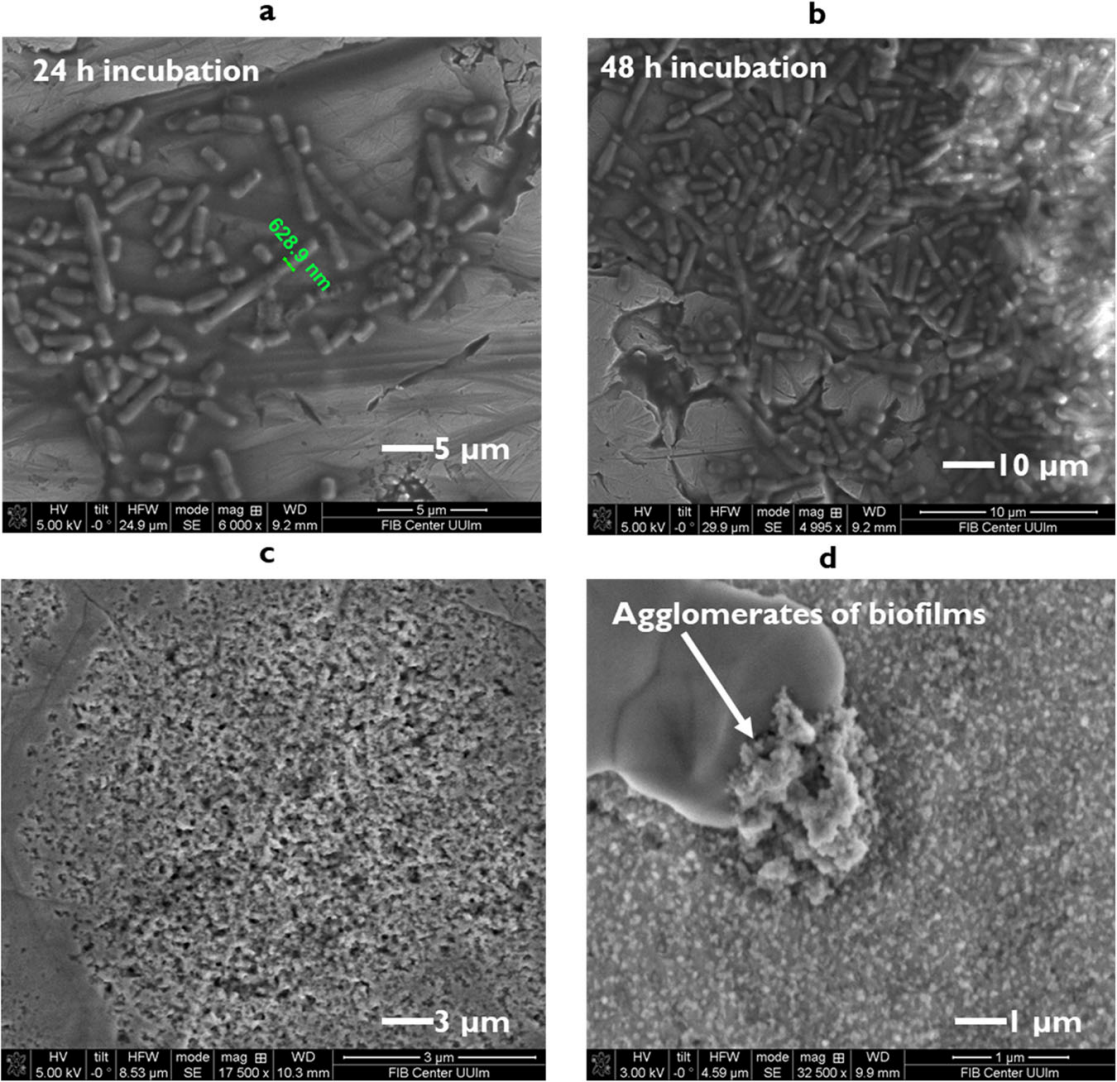
Scanning electron microscopy images of *L. parabuchneri* DSM 5987 biofilms grown at stainless steel surfaces. Biofilm-forming *L. parabuchneri* cells: **a** after 24 h of incubation; **b** after 48 h of incubation; **c** after 72 h of incubation, and **d** by exemplary agglomerates embedded into EPS and adherent to the surface. 1–10 μm scale bars are shown on micrographs.

We also investigated *L. parabuchneri* biofilm formation at an industry-relevant surface, i.e., stainless steel. Biofilms forming at the surface of food processing equipment represent a reservoir of histamine-producing bacteria, and thus, a major source of contamination, e.g., during post-ripening of cheeses. To observe the biofilms developed at stainless-steel coupons, the method of Kubota et al.^89^ was followed with some modifications. Scanning electron microscopy (SEM) images of the biofilm formed were collected at similar increments as for the IR studies, i.e., after 24, 48, and 72 h of incubation. In relation to early-stage biofilms formed, the first attached microcolonies on stainless steel surface are shown by SEM imaging (Supplementary Fig. 4). Clusters of cells evidently forming a biofilm appear embedded in EPS adherent to the coupons (Fig. 10), which confirms similar behavior as observed during the IR spectroscopic investigations at the ZnSe ATR surface and the scenarios derived from grated cheese manufacturing environments^9^. The average number of cells adhered to the stainless-steel coupons was >10^5^ CFU/cm^2^. The radius of *L. parabuchneri* cells with typical elongated shape after 24 h of incubation was around 630 nm (Fig. 10a).

The histamine producing species *Lentilactobacillus parabuchneri* adheres to the stainless-steel coupons, which is the most commonly used surface in the cheese processing and manufacturing industry. In this study, it is elaborated the molecular changes of biofilms grown in common surface of real applications like stainless steel compared to ZnSe surface. While compact aggregates of biofilms were formed within the first 24 h as indicated by SEM micrographs^10^ (Fig. 10a), biofilm attachment at the ZnSe waveguide surface during different times of incubation provides only bulk information and was focused on revealing molecular changes during biofilm formation via IR-ATR spectroscopy and multivariate data analysis^90^. Instead, SEM imaging reveals structural arrangements, yet no molecular information, i.e., aggregates of cells at early stages of biofilm formation were not homogenously attached to the surface of the coupons, and immature biofilms revealed micro-clustered cells^91^. The biofilm morphology of early biofilms is indicative of early-to-intermediate biofilm formation prior to significant EPS production, which was precisely noted at a molecular level via IR-ATR real-time monitoring. The majority of biofilms observed during SEM studies revealed an elongated shape during the initial 24 h, while irregular shapes with branched patterns of the colonies were characteristic for more mature stages at 48 and 72 h of incubation. Again, the observed changes were associated with two main factors derived from the IR-ATR studies at those periods: nutrient concentration and EPS formation^25^, which correlate well with the SEM observations. The trajectory of the EPS concentration during 72 h (Fig. 5c) may indeed be explained by communication via quorum sensing of *L. parabuchneri* cells resulting in the formation of microcolonies (Fig. 4, supplementary information).

Several characteristic IR bands are also sensitive to structural changes of microbial cells including intra- and intermolecular interactions via H-bonding patterns, membrane arrangements, lipid–protein interactions, and conformational state changes of proteins. In addition, the physical state of the sample including hydration or aggregation, interaction with ions, etc. also affects these signatures^71^, while the EPS composition determines the ability inducing cell aggregation during biofilm maturation^33^.

The mature stages of biofilm growth observed via SEM images of biofilms at stainless steel coupons (Fig. 10c) are dominated by cell division rather than free microbial cell attachment^92,93^. During real-time IR-ATR monitoring, the increase of characteristic bands within the IR spectra at mature stages appear proportional to the increase of biofilm thickness, as shown in Figs. 5c and 6. The biofilm matrix covered the entire ATR waveguide surface. When supplied with fresh MRS media, the metabolically active *L. parabuchneri* layers contribute to the nutrient gradient resulting in the periodic replacement of old bacteria layers. The increase of proteins reported in the IR spectra by the increase of both amide groups and the constant increase of EPS gives rise to the formation of substantial biofilm mass. Irregular biofilm attachment indicated by intensity fluctuations of some indicative IR bands results from the fact that top layer of bacteria in close proximity to the ATR waveguide acts as a temporary barrier between the nutrient-rich MRS media phase and the gel-like biofilm phase^37^. Hence, *L. parabuchneri* biofilms at advanced stages appear as patterned elongated piles. The optical micrographs of L. parabuchneri 7 days old biofilms on top of ZnSe surface revealed the structural heterogeneity of biofilms (Supplementary Fig. 5). Furthermore, the variations in contrast evident in the SEM images distinctly indicate variations in the layer thickness and agglomerate dimensions of stable biofilms. Mature biofilms at stainless steel surfaces after 72 h of formation cover first-layer biofilms, which is associated with extensive EPS formation^25^. The fact that the biofilms do not grow as a planar surface layer is related to the presence of polysaccharides remaining from the first layers of the initial biofilms^94^. Several areas covered by cells within the young biofilm layers evident in the SEM images confirm that the matrix is indeed produced by *L. parabuchneri* cells. In turn, the molecular compositional changes observed via the IR-ATR studies prove that the thin biofilm layer observed during SEM imaging is indeed augmented via EPS-mitigated attachment. Summarizing, the combination of these two analytical methods—and in future additional imaging and/or molecular spectroscopic techniques—provides an in-depth understanding on biofilm formation and may clearly be expanded beyond studies on *L. parabuchneri*, yet, starting to unravel the mechanisms of *L. parabuchneri* biofilm formation in molecular detail via IR-ATR spectroscopy in combination with SEM imaging herein for the very first time.

The performance of the crystal violet staining method (CV) as a biofilm-biomass quantification procedure^89^ allowed to make the appropriate classification of the biofilm formation ability of the *L. parabuchneri DSM 5987* strain. Biofilm production is expressed following cut-off values, i.e., the mean ± SD of the optical density (OD) of three replicates was calculated for the strain. A negative control was made to compare with the absorbance of *L. parabuchneri DSM 5987* (OD_600_ = 1.4 ± SD). The OD of the control (ODc = 0.3 ± 0.09) was used to classify the strain in moderate producer with the following cut-off values obtained: ODc = 0.57 < ODc × 2 = 1.14 < OD_DSM 5987_ < ODc × 4 = 2.28. *L. parabuchneri DSM 5987* strain aggregates into micro clusters, chains of undivided cells, and eventually mature biofilms that are of concern for surfaces used in food industry.

Moderate biofilm producers remain a problem in food industries, as they act as a persistent source of microbial contamination that leads to food spoilage. The high proteolytic enzyme activity associated with *L. parabuchneri* DSM 5987 strain has increased the potential for biogenic amine, i.e., histamine production. The matrix formation via biofilms facilitates the surface attachment as the microbial colony matures, and the biofilm-forming capacity indeed may serve as an indicator of the potential degree of contamination in dairy products. Hence, studies on a variety of *Lactobacillus* strains based on the presented fundamental findings will enhance our knowledge on lactic acid bacterial biofilms and devise potential mitigation strategies.

## Discussion

The chemistry of *Lactobacillus* biofilms is based on a suite of signaling compounds that are coordinated in activity via quorum sensing mechanisms, i.e., diffusible signal molecules regulating multi-cellular behavior. Quorum sensing promotes adherence to substrates, improves access to nutrients, and triggers the formation of biofilms^95,96^. The formation of complex multi-cellular communities requires cell-to-cell communication via extracellular messenger molecules (i.e., extracellular matrix components) or direct cell-to-cell contact. In addition, small molecules regulating multi-cellular behavior may also serve as signals similar to concerted signaling cascades within single-species communities^97^. Biofilms with a low growth rate frequently induce the production of an extracellular matrix as a major component of microbial biofilms detectable by IR spectroscopy. Within the first hours of *L. parabuchneri* biofilm formation, the IR signals of the EPS matrix were distinctly noticeable. The EPS matrix has a protective function and conserves a favorable microenvironment. This leads to the formation of so-called microcolonies, and microorganisms start to proliferate sending chemical signals for communication among bacterial cells^98^. Thus, attached microorganisms reproduce, and the complexity of the community as well as the overall density increases. Quorum sensing, gene transfer, and persisted development are processes that are essential for the community to proliferate with the biofilm environment. In parallel, the biofilm is approaching a state of maturity, and conditions at the base layer become increasingly anaerobic if the biofilm reaches a thickness of ~10–25 µm with a variety of factors affecting the viability of the biofilm^99,100^.

The present study confirms real-time infrared spectroscopy as a useful analytical tool for in situ and non-destructively monitoring the evolution of *L. parabuchneri* biofilms. IR-ATR spectroscopy provides real-time data on the vibrational signatures of the main constituents involved in biofilm formation and maturation in molecular detail during the extended unperturbed observation periods. The spectral range between 1700 and 600 cm^‒1^ is of particular interest providing information on molecular processes via significant changes of the amide I and II bands representing the protein signature of the biomass, nucleic acids, and lipids along with the characteristic features of extracellular polymeric substances indicating formation, growth, and maturation of the biofilm. Multivariate data analysis and classification strategies were applied facilitating the multiparametric analysis of the molecular processes involved in biofilm formation, as well as the classification of biofilms by age. Last but not least, first studies derived from these findings were transferred to *L. parabuchneri* biofilm formation at biotic and abiotic surfaces with a particular focus on stainless-steel surfaces relevant in food processing and dairy industries.

While only few studies report on the biofilm formation mechanisms and associated food spoilage by histamine-producing *Lactobacillus* species, to the best of our knowledge this is the first comprehensive study monitoring molecular details during biofilm formation via IR-ATR spectroscopy. In future, the integration of additional orthogonal sensing techniques will facilitate monitoring additional biochemical parameters and molecular changes (e.g., oxygen concentration, pH changes, etc.) associated with the observed metabolic patterns for completely unraveling the molecular processes occurring within microbial biofilms.

## Methods

### Bacterial strain, culture media, and growth conditions

The DSM 5987 strain of *L. parabuchneri* strain was provided by the Leibniz Institute DSMZ (Braunschweig, Germany) and requires microaerophilic growth conditions or anaerobic cultivation within oxygen-free media. *L. parabuchneri* cells were grown and maintained in a nutritionally rich medium characteristic to species of lactobacilli, i.e., Man de Rogosa Sharpe-MRS broth^101^ at 30 °C. DSMZ medium 11 was freshly prepared by the addition of MRS components (10 g L^‒1^ tryptic digestive casein peptone, 10 g L^‒1^ meat extract, 5 g L^‒1^ yeast extract, 20 g L^‒1^ glucose, 1 g L^‒1^ polysorbate 80, 2 g L^‒1^ dipotassium phosphate K_2_HPO_4_, 5 g L^‒1^ sodium acetate, 2 g L^‒1^ tri ammonium citrate, 0.2 g L^‒1^ magnesium sulfate heptahydrate MgSO_4_ x 7 H_2_O and 0.05 g L^‒1^ manganese sulfate monohydrate MnSO_4_ x H_2_O) homogenized with deionized Milli-Q water, and adjusted to pH 6.3. The overnight inoculated samples were added on an anaerobic workstation (Forming gas—95% N_2_ + 5% H_2_) and then incubated at 30 °C for 24, 48, and 72 h. To test the capacity of the strain for histamine production, the cultures were supplemented with 5 mM Histidine^9^. The strains were isolated at −80 °C in MRS with 10% (w/v) sterile glycerol.

### *Lactobacillus* suspensions for biofilm formation

The harvesting of bacteria for freshly prepared *L. parabuchneri* suspensions was followed by an overnight culture sample preparation. Strains of *L. parabuchneri* were inoculated in 50, 100, 200, and 500 mL of MRS medium, and the bacteria were grown overnight for 24 h. Once the cells reached the end of the exponential growth phase, the samples were centrifuged (4000 rcf, Eppendorf Centrifuge 5430, 10 min). Thereafter, the supernatant was discarded, and the residue was resuspended in sterile fresh MRS media. For monitoring the bacterial concentration and bacterial growth, the optical density (OD) of the bacterial suspensions was examined at 600 nm via UV-VIS spectrophotometer (Specord S600, Analytik Jena AG, Germany). Spectrophotometric measurements of the developing turbidity at hourly intervals were correlated with the increasing number of cells. The bacterial concentration of sub-cultures reached an OD_600_ of ~0.25–2.5 depending on the overnight culture type. Prior to the inoculation into the flow system, the media were purged for 15 min with nitrogen to ensure oxygen-free conditions. To determine the capacity of the isolated strain for producing histamine, the strain was supplemented with 5 mM L ( + )-Histidine (Sigma Aldrich, Chemie GmbH, Germany) for 24 h at 30 °C. In Supplementary Fig. 1 is shown the flow-chart of the optimized protocol for microaerophilic cultivation of *Lactobacillus parabuchneri* bacterial cultures as a facultative microorganism at oxygen-free conditions.

### Oxygen monitoring ensuring constant biofilm growth

The microaerophilic environment for constant biofilm growth allows a more precise evaluation of bacterial behavior. The MRS medium was purged with a mix of N_2_/CO_2_ gas for 15 min via silicone tubes in flow mode. Afterward, the oxygen levels were monitored using a retractable fiberoptic oxygen microsensor based on luminescence quenching (OXR50, Pyroscience, Aachen, Germany)^102^. The sensor was calibrated via a recommended two-point calibration using an oxygen-free and an air-saturated aqueous solution. Growth of the initial biofilm was not affected by minimal amounts of dissolved oxygen (Table 2).

### Biofilm growth

Biofilm monitoring was reproducibly performed with the following procedural scheme: (i) disinfection of the entire assembly by flushing with 4% solution of Korsolex (VWR Germany) for 20 min at 2.2 mL/min; sterile MRS broth solution for 120 min at 0.7 mL/min to create a conditioning film; (iv) introduction of the *L. parabuchneri* suspension for 6 h at 0.7 mL/min; (v) finally, providing sterile MRS media at 0.7 mL/min for 24 h up to several days for extended observation periods. Both the MRS solution and MilliQ water were autoclaved prior to use (121 °C, 15 min, 3 bar, Systec VE-150).

### Infrared attenuated total reflection spectroscopy

**Fig. 11:**
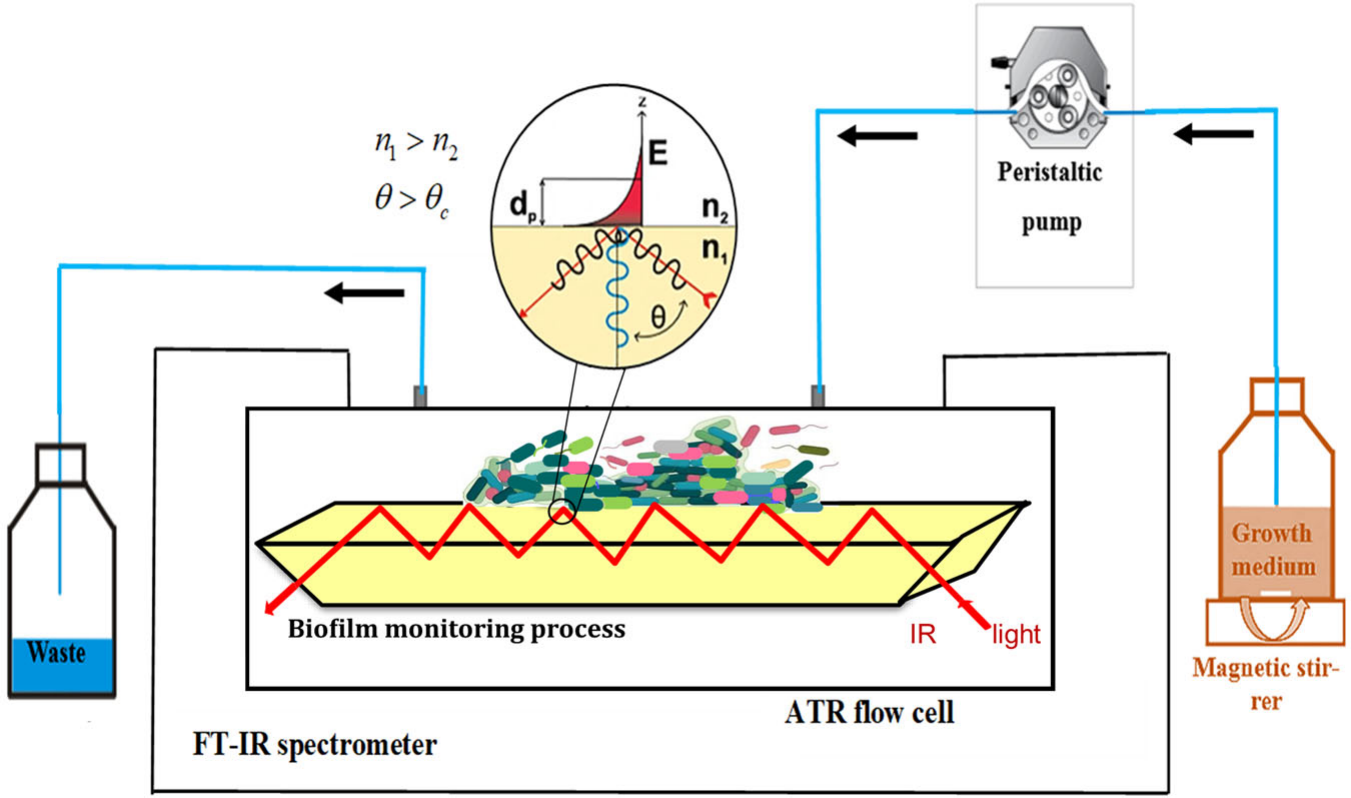
Custom IR-ATR flow cell concept. The continuous flow assembly was equipped with a ZnSe ATR waveguide providing 6 internal reflections for monitoring biofilm formation and evolution via evanescent field absorption spectroscopy.

For in situ monitoring *of* bacterial biofilms, an advanced IR-ATR continuous flow assembly attached to a Bruker Alpha I Fourier transform infrared (FTIR) spectrometer (Bruker Optics, Ettlingen, Germany) was developed (Fig. 11). The flow cell encloses a six-reflection ZnSe ATR-crystal (dimensions ~48 × 5.5 × 4 mm at the top side), by the customized removable top plate which contains luer-lock connectors for flowing solutions through the cell^37^.

This custom-made flow cell cover was designed and fabricated from PEEK and equipped with a 3D-printed O-ring gasket made from PLA. The cell volume was ~0.70 mL. The assembly was equipped with a peristaltic pump (Alitea, Sweden); silicon tubing was connected to the system via Leuer-lock connectors.

During biofilm formation, IR-spectra were recorded in the range 4000–400 cm^‒1^ at a spectral resolution of 2 cm^‒1^. 100 scans were averaged for each spectrum. All interferograms were Fourier transformed using the Mertz phase correction mode and the Blackman-Harris three-term apodization function. Water vapor subtraction and baseline correction were performed for all obtained IR-spectra. The deconvolution of IR-spectra data was performed using spectra normalized via the sum of Gaussian curves. The spectrum of deionized water was used as the background prior to MRS introduction into the flow system. After 2 h of MRS conditioning film formed, the collection of the background spectrum is done for *L. parabuchneri* biofilm dataset to minimize the water contributions to the overall spectra. Recording of spectra and all spectral calculations were performed using the OPUS 8.1 software (Bruker Optics, Ettlingen, Germany). For further processing, the IR-spectra were converted to Excel datasets using the EssentialFTIR software package (Operant LLC, Madison/USA), and the OriginLab software package (OriginLab Corp., Massachusetts/USA). During extended biofilm monitoring experiments, IR-spectra were recorded every 10–15 min at 22 ± 1 °C in an air-conditioned laboratory. Multivariate data analysis was performed using MATLAB software R2018b (The MathWorks, Inc., United States).

### Electron microscopy imaging

To further analyze the biofilms developed at stainless-steel coupons, the method of Kubota et al. was followed with some modificationss^70^. Briefly, V_2_R stainless steel coupons (0.8 mm thickness) were incubated at 30 °C for 24, 48, and 72 h with bacteria inoculated in MRS, then rinsed with PBS buffer, and fixed in 2.5% glutaraldehyde. After dehydration with a graded series of acetone solutions (50–100%), the samples were dried under Argon flux, coated with Pt (SDC 005 sputter coater), and subject to scanning electron microscopy (SEM) studies using a dual-beam FIB/SEM system (Quanta 3D FEG, FEI Company, Eindhoven, NL).

### Crystal violet (CV) staining biofilm quantification

*L. parabuchneri* biofilms produced on polystyrene surfaces were prepared based on the method of Diaz et al.^9^ with some modifications. The overnight bacterial dilutions (200 µL) were cultivated on 96-well polystyrene microtiter plates along with controls in 8 wells by sterile MRS media and incubated at 30 °C. Crystal violet staining was used for biofilm biomass quantification^89^. After 48 h of incubation, the wells were rinsed twice with PBS buffer, air-dried for 30 min at room temperature. Biofilms formed were stained with 250 µL of 0.1% (w/v) crystal violet in dH_2_O for 30 min. The non-bound dye was removed and rinsed three times with 300 mL of dH_2_O. The bound dye with biofilm was extracted using 200 mL of acetone/ethanol (80/20) and absorbance was measured at 600 nm using an UV-Vis spectrometer. Biofilm production ability was expressed using cut-off values as described in Diaz et al.^9^. The mean ± SD of the optical density (OD) of three replicates was calculated. The cut-off value between biofilm-producers and non-producers was defined as the mean of the negative controls (ODnc) plus three SDs (ODc). The strains were then classified into the following categories:

$${{{\mathrm{ODc}}}} \,<\, {{{\mathrm{OD}}}} \le 2 \times {{{\mathrm{ODc}}}} = {{{\mathrm{weak}}}}\,{{{\mathrm{biofilm}}}}\,{{{\mathrm{producer}}}}$$

$$2 \times {{{\mathrm{ODc}}}} < {{{\mathrm{OD}}}} \le 4 \times {{{\mathrm{ODc}}}} = {{{\mathrm{moderate}}}}\,{{{\mathrm{biofilm}}}}\,{{{\mathrm{producer}}}}$$

$${{{\mathrm{OD}}}} \,>\, 4 \times {{{\mathrm{ODc}}}} = {{{\mathrm{strong}}}}\,{{{\mathrm{biofilm}}}}\,{{{\mathrm{producer}}}}$$

### Reporting summary

Further information on research design is available in the Nature Research Reporting Summary linked to this article.

## Supplementary information

Supplementary Material

Reporting Summary

## Data Availability

All data measured and analyzed during this study are included in the paper and its supplementary information file. Additional data are available from the corresponding author upon reasonable request.
