# Supplementary material for: In situ monitoring of Lentilactobacillus parabuchneri biofilm formation via real-time infrared spectroscopy
Source: NPJ Biofilms Microbiomes. 2022 Nov 19;8:92. doi: 10.1038/s41522-022-00353-5 (PMC9675856; doi:10.1038/s41522-022-00353-5)
Supplement: Supplementary file 1 — Supplementary Material [file 41522_2022_353_MOESM1_ESM.pdf]

Supplementary Information

In situ monitoring of *Lactobacillus parabuchneri* biofilms via real-time infrared spectroscopy

Diellza Bajrami[1], Stephan Fischer[2], Holger Barth[2], Mária A. Sarquis[3], Victor M. Ladero[3], Maria. F. García[3], Maria. C. Sportelli[4], Nicola Cioffi[4], Christine Kranz[1] and Boris Mizaikoff[1, *]

1 Institute of Analytical and Bioanalytical Chemistry, Ulm University, Albert Einstein-Allee 11, 89081 Ulm, Germany; diellza.bajrami@uni-ulm.de (D.B.); christine.kranz@uni-ulm.de (C.K.); boris.mizaikoff@uni-ulm.de (B.M.)

2 Institute of Pharmacology and Toxicology, Ulm University Medical Center, Albert Einstein-Allee 11, 89081 Ulm, Germany; stephan-1.fischer@uni-ulm.de (S.F.); holger.barth@uni-ulm.de (H.B.)

3 Dairy Research Institute, IPLA-CSIC, Paseo Rio Linares s/n, 33300 Villaviciosa, Spain; agustina.sarquis@ipla.csic.es (M.A.S.); ladero@ipla.csic.es (V.M.L.) mfernandez@ipla.csic.es (M.F.G.)

4 Chemistry Department, University of Bari "Aldo Moro", V. Orabona, 4, 70126, Bari, Italy; maria.sportelli@uniba.it (M.C.S.); nicola.cioffi@uniba.it (N.C.)

* Correspondence: Prof. Dr. Boris Mizaikoff (boris.mizaikoff@uni-ulm.de; Tel.: +49-731-50-22750)

This PDF file includes:

Supplementary notes 1-4
Supplementary discussion
Supplementary Figures 1-5
Supplementary Table 1
Supplementary References

Supplementary note 1

Experimental strategy for *L. parabuchneri* cultivation

Lactic acid bacteria are Gram positive aerotolerant microorganisms that synthesize lactic acid as the main product of sugar fermentation. The culture media and the main fermentation conditions requires a nutritive media rich with carbon source[1]. Mann de Rogosa Sharpe broth has been used as an oxygen free media for cultivation of the *L. parabuchneri* DSM 5987 isolates. To test the capacity of strain for Histamine producing, the cultures have been supplemented with 5mM Histidine[2]. By the scheme you can see the matrix of the overnight inoculated samples incubated on 30°C, 24-72h. After that the supernatant was discarded and resuspended on fresh MRS media, stirred for 2 h and afterwards measuring the OD. Before the inoculation to the flow system, the media was flushed by nitrogen gas (degassing process) and the oxygen level were monitored using an O_2 microsensor.

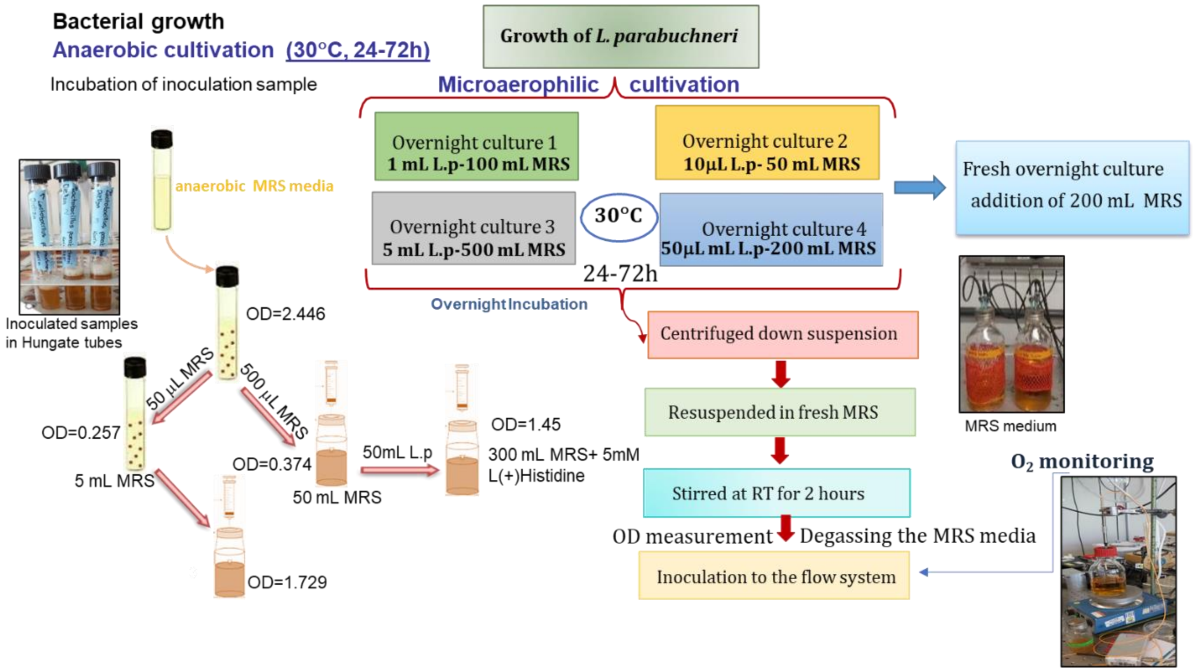

Supplementary Figure 1. Experimental strategy for *L. parabuchneri* cultivation.

Supplementary note 2

The process of *L. parabuchneri* biofilm formation at the ATR waveguide surface with five distinct stages of biofilm evolution

Development of lactobacillus biofilms leads toward series of morphological changes on the substrate[3,4]. The adhesion to surface is dependent physicochemical properties of the abiotic and biotic surfaces like: pH, hydrophobicity, surface texture, temperature, etc[5]. The five proposed stages of biofilm formation are:
1) initial attachment of cells at the surface;
2) transport and attachment of bacterial cells towards the surface;
3) early development of biofilms indicated by EPS production and proliferation into microcolony units;
4) maturation of the biofilm architecture, and
5) dispersal of mature clusters and cell detachment (schematically illustrated in Figure 2).
Many cells can detach from the surface and come back to the planktonic lifestyle, spreading out to start a new cycle of life. Extracellular polymeric matrix has the key role to adhesion ability and strength the bond between bacteria and the substrate.

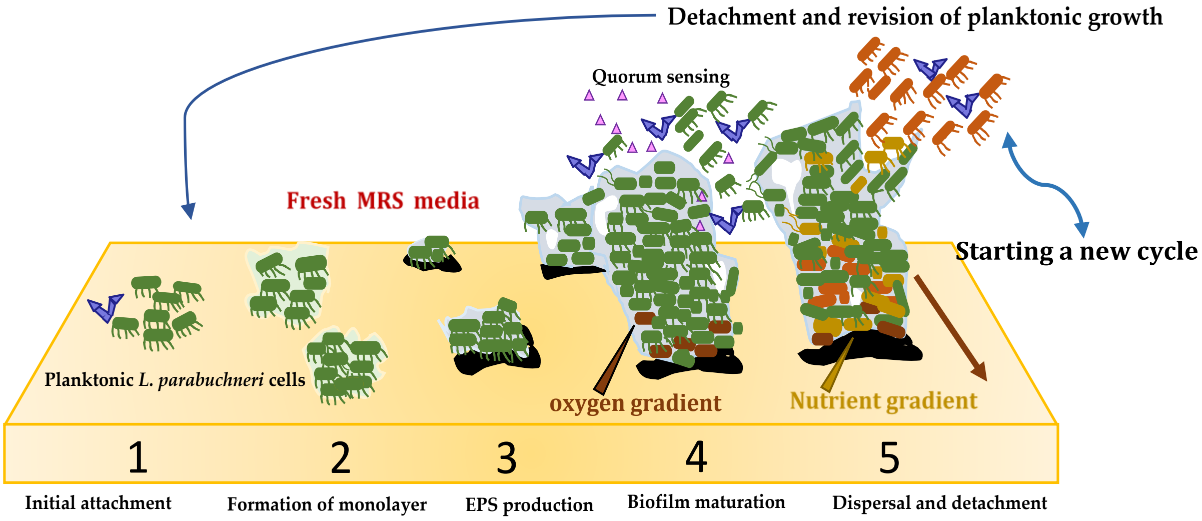

Supplementary Figure 2. Scheme of the five stages of *L. parabuchneri* biofilm development regulated by quorum sensing.

Supplementary note 3

IR-spectroscopic characterization of the individual components of de Man Rogosa and Sharpe medium (MRS)

IR-ATR spectroscopy with the Alpha I spectrometer of the individual constituents of MRS showed the characteristic spectra in the spectral region from 1800 – 900 cm^{-1}. The peaks of the individual constituents were integrated and averaged thereafter served as basis for the cumulative impulse fit demonstrated in the manuscript. Spectra were recorded using the same Alpha spectrometer with Platinum Single bounced Diamond Crystal in 2 cm^{-1} resolution and 100 average scans. The characterization of the MRS growth medium is crucial to chemically analyze *L. parab.* adsorption and biofilm formation in molecular detail and the cumulative impulse fit of the spectra of de Man Rogosa and Sharpe medium (MRS) is fundamental for the analysis of *L. parab.* biofilms in this culture medium.

a

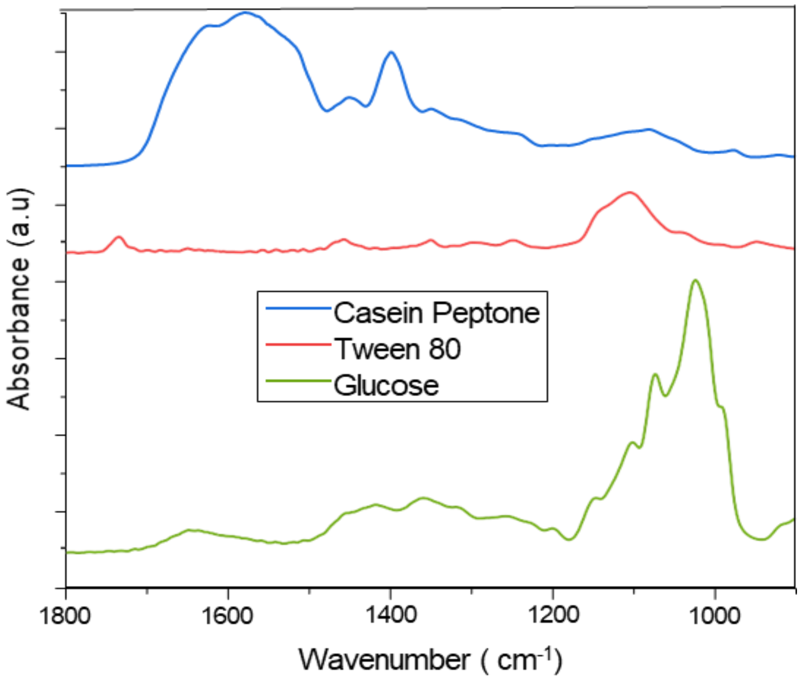

b

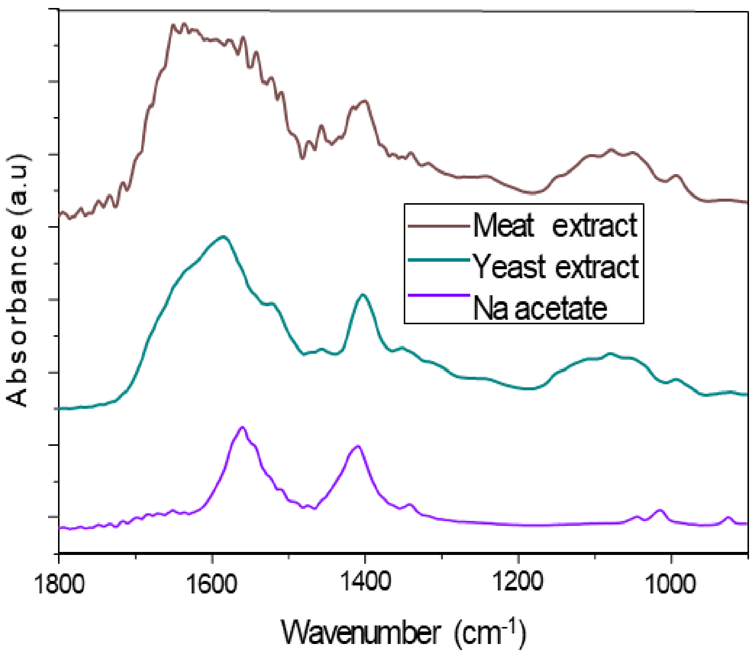

c

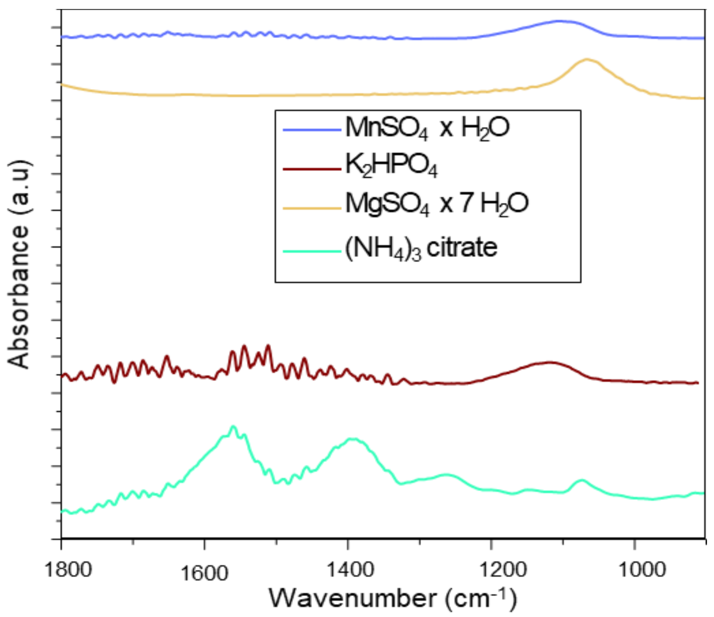

Supplementary Figure 3. **IR-ATR spectra of the individual constituents of MRS in the spectral region from 1800 – 900 cm^{-1}** (see the color label for each component).

Supplementary note 4

Scanning electron microscopy of *L. parabuchneri* biofilms

a b

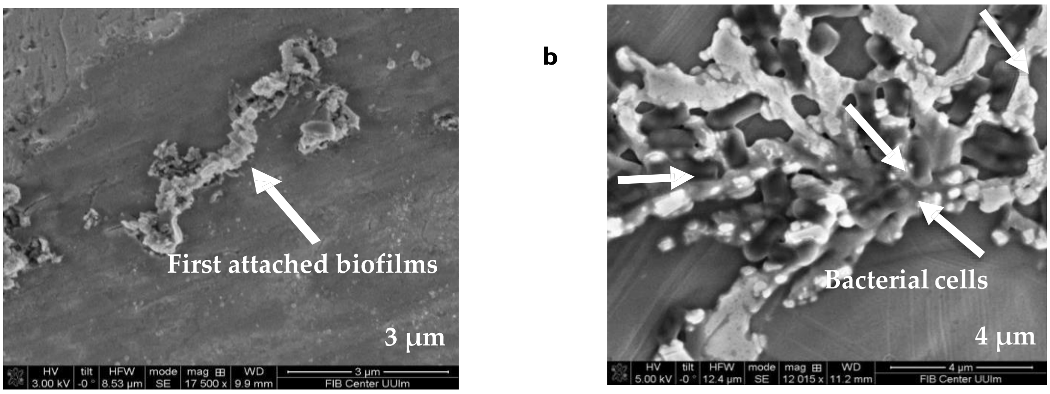

Supplementary Figure 4. Scanning electron photomicrographs of *Lactobacillus parabuchneri* biofilms adhered to stainless steel coupons within a) the first attached microcolonies formed on surface and b) bacterial cells well adaption.

Supplementary discussion

Optical microscopy of *L. parabuchneri* biofilms

The characterization of the chemistry and architecture of biofilms can be evaluated by the combination of IR-ATR spectroscopy and light microscopy and therefore achieve the information on the region which is restricted by evanescent wave depth penetration [6]. For 7 days inoculation on the flow ATR system, the ZnSe surface has become highly colonized by *L. parabuchneri* bacteria creating high density biofilms with different heterogeneity types. These images show uniform coverage of ZnSe surface by *L. parabucneri* biofilms. Figure 5 a show the biofilms visualized after 24 h (1 day), figure 5 b shows the biofilm attached to waveguide surface after 72 h (3 days) and figure 5 c shows the mature biofilm after 168 h (7 days).

For 7 days of inoculation within the IR-ATR assembly, the ZnSe surface has become highly colonized by *L. parabuchneri* bacteria creating high-density biofilms with spatially different heterogeneities. Optical microscopy is a basic technique to track the structural heterogeneity of biofilms. The optical micrographs prove the coverage with biofilms at the waveguide surface. These images reveal biofilm structures adherent to the ZnSe surface in small colonies of cells after 24 h (Fig 5 a), whereby the number of *L. parabuchneri* cells that were near the attached 'pioneering cells' was higher than those observed close to the initially uncovered zones of the ZnSe substrate. A uniform coverage of the ZnSe surface by *L. parabuchneri* biofilms was observed after 3 days (Fig 5.b) of real-time monitoring with the addition of fresh nutritive media every 16 h. The aged biofilms after 7 days (Fig 5.c) reveal an increased thickness of the biofilm enriched with nutrients for a week. Microscopic images were captured with Zeiss Microscope (Zeiss, Germany) and defined by the image analysis micro software.

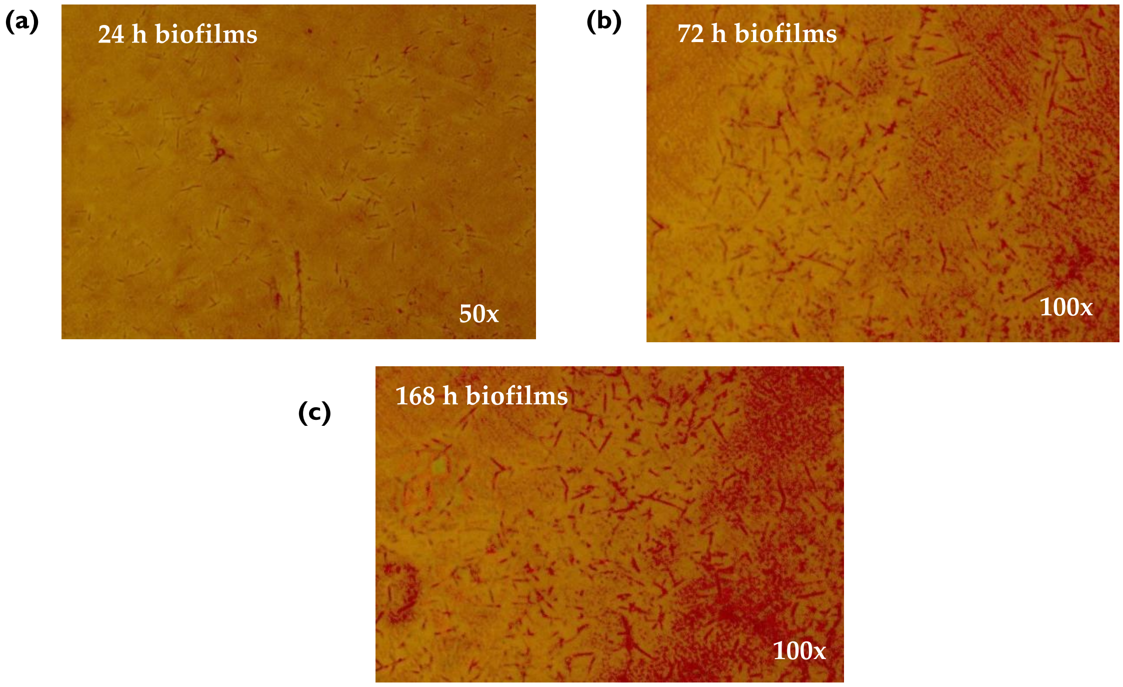

Supplementary Figure 5. Optical micrograms of *L. parabuchneri* bacteria that have attached the surface of ZnSe crystal during 7 days of flow-through IR measurements whereby changes in biofilm parameters that are resolved and quantified by light microscopy and computer-controlled image analysis can be related to surface area-averaged chemical changes at the biofilm-substrate interface.

Supplementary Table 1. Fundamental infrared vibrational band assignments of the 3000 – 500 cm^{-1} spectral region of the IR-ATR spectrum of *L parabuchneri* bacterial cells and biofilm (υ: stretching, δ: bending, τ: twist, a: antisymmetric, s: symmetric, ARN: Ribonucleic acid; AND: Deoxyribonucleic acid and LPS: lipopolysaccharides)[7–9].

Wavenumber (cm^{-1})	Assignment	Principal constituents	Cellular components
2961	υ_a CH_3	Fatty chains	Cell membrane
2925	υ_a CH_2	Fatty acids	Cell membrane
2897	υCH tertiary	Fatty acids	Cell membrane
2874	υ_sCH3	Fatty acids	
2854	υ_s CH2		
1736	υC=O	Esters from lipids	Cell membrane
1713	υC=O	Esters, carboxylic acids	Nucleoid, ribosomes
1700–1580	C=O C=N C=C NH	ADN/ARN bases	Nucleoid, ribosomes
1693–1627	Amide I (υ C=O coupled with N-H δH_2O	Proteins, water (1640 cm^{-1})	Membranes, cytoplasm, flagella, pili, ribosomes
1568–1531	Amide II (δN-H coupled with C-N)	Proteins	Membranes, cytoplasm, flagella, pili, ribosomes
1468–1455	δCH_2, δ_a CH_3	Lipids	Membranes
1400	υ_s COO^-	Amino acids, fatty acid chains	Capsules, peptidoglycan
1350; 1281	τCH_2, ρCH_2Amide III (υC-N coupled with δN-H)	Fatty acid chains, proteins	Membranes, nucleoid, ribosomes
1238	PO_2^-	Phosphodiester, phospholipids, LPS, nucleic acids, ribose	
1220	υC-O-C	Polysaccharides	
1200-900	υC-O, υC-C,δC-O-C	Polysaccharides	Capsule, storage inclusions
1172; 1153	υ_sC-OH, υC-O	Proteins, carbohydrates, esters	
1118	υ_sCC	ARN, ADN	Nucleoid, ribosomes
1086	υ_s PO_2^-	Phosphodiester, phospholipids, LPS, nucleic acids	Membranes, nucleoid, ribosomes
1058	υ_sC-O-C, υ_s P-O-C	Polysaccharides	Capsule, peptidoglycan
1041	υO-H coupled with δC-O	Polysaccharides	Capsule, peptidoglycan, cell wall
1026	CH_2OH	Carbohydrates	Storage inclusion
993		Ribose skelet (ARN)	Ribosomes
970	υC- C, υP-O-P	RNA backbone	Ribosomes
900-500	–	"true" fingerprint region	Bands not assigned to specific functional groups

Supplementary References

1. Sánchze, Ó. J., Barragán, P. J. & Serna, L. Review of Lactobacillus in the food industry and their culture media. *Rev. Colomb. Biotecnol.* **21**, 63–76 (2019).
2. Sumner, S. S., Speckhard, M. W., Somers, E. B. & Taylor, S. L. Isolation of histamine-producing Lactobacillus buchneri from Swiss cheese implicated in a food poisoning outbreak. *Appl. Environ. Microbiol.* **50**, 1094–1096 (1985).
3. Stoodley, P., Sauer, K., Davies, D. G. & Costerton, J. W. Biofilms as complex differentiated communities. *Annu. Rev. Microbiol.* **56**, 187–209 (2002).
4. Salas-Jara, M., Ilabaca, A., Vega, M. & García, A. Biofilm Forming Lactobacillus: New Challenges for the Development of Probiotics. *Microorganisms* **4**, 35 (2016).
5. Donlan, R. M., Costerton, J. W., Donlan, R. M. & Costerton, J. W. Cmr.15.2.167-193.2002. **15**, (2002).
6. Suci, P. A., Siedlecki, K. J., Palmer, R. J., White, D. C. & Geesey, G. G. Combined light microscopy and attenuated total reflection fourier transform infrared spectroscopy for integration of biofilm structure, distribution, and chemistry at solid-liquid interfaces. *Appl. Environ. Microbiol.* **63**, 4600–4603 (1997).
7. Oust, A., Møretrø, T., Kirschner, C., Narvhus, J. A. & Kohler, A. FT-IR spectroscopy for identification of closely related lactobacilli. *J. Microbiol. Methods* **59**, 149–162 (2004).
8. Ojeda, J. J. & Dittrich, M. Fourier transform infrared spectroscopy for molecular analysis of microbial cells. *Methods Mol. Biol.* **881**, 187–211 (2012).
9. McWhirter, M. J., Bremer, P. J. & McQuillan, A. J. Direct infrared spectroscopic evidence of pH- and ionic strength-induced changes in distance of attached Pseudomonas aeruginosa from ZnSe surfaces. *Langmuir* **18**, 1904–1907 (2002).
